# Synaptonemal complex SUMOylation is maintained by Nup60-dependent docking of Ulp1 at the nuclear periphery

**DOI:** 10.1016/j.celrep.2026.117539

**Published:** 2026-06-11

**Authors:** Rahel Wettstein, Grant A. King, Adrian Henggeler, Madison E. Walsh, Cyrus T. Ruediger, Elçin Ünal, Joao Matos

**Affiliations:** 1 Max Perutz Labs, Vienna BioCenter, 1030 Vienna, Austria; 2 University of Vienna, Vienna, Austria; 3 Institute of Biochemistry, ETH Zürich, 8093 Zürich, Switzerland; 4 Department of Molecular and Cell Biology, University of California, Berkeley, Berkeley, CA 94720, USA; 5 California Institute for Quantitative Biosciences (QB3), University of California, Berkeley, Berkeley, CA 94720, USA; 6 Center for Computational Biology, University of California, Berkeley, Berkeley, CA 94720, USA; 7 These authors contributed equally; 8 Lead contact

## Abstract

The nuclear pore complex (NPC) basket has been implicated in regulating meiotic recombination, but the underlying mechanism remained elusive. Here, we show that most basket subunits are required for controlled crossing-over in budding yeast. Central to this function, the nucleoporin Nup60 anchors the SUMO protease Ulp1 at the nuclear periphery, thereby protecting the synaptonemal complex (SC) protein Ecm11 from premature deSUMOylation. Unscheduled dissociation of Ulp1 from the NPC impairs Ecm11 SUMOylation, disrupts synapsis, elevates crossovers, and compromises gamete viability. Remarkably, engineered tethering of Ulp1 to the NPC restores SC integrity and recombination control in basket mutants. We further show that Polo-like kinase Cdc5 remodels SUMO homeostasis at the prophase I-metaphase I transition, triggering partial Ulp1 release from the NPC and phosphorylating the SUMO ligases Siz1 and Siz2. These findings uncover how nucleoporins, SUMO enzymes, and kinase signaling cooperate to coordinate SC dynamics with crossover control, safeguarding meiotic genome transmission.

## In brief

Wettstein et al. identify the NPC as a spatial regulator of SUMO proteolysis in meiosis. By restricting the SUMO protease Ulp1, the NPC shapes SUMO dynamics that control synaptonemal complex behavior and crossing over, with Cdc5 targeting SUMO proteases and ligases at the meiosis I onset to remodel this landscape.

## Graphical Abstract

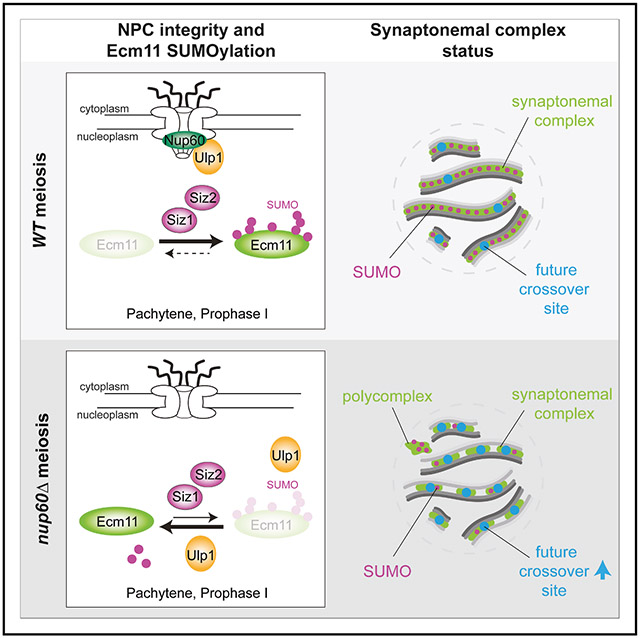

## INTRODUCTION

The nuclear pore complex (NPC) is the defining gateway between the cytoplasm and the nucleus. Beyond its canonical role in nucleocytoplasmic transport,^1,2^ the NPC has now emerged as a multifunctional structure regulating gene expression, genome stability, and chromatin organization^3-10^—functions largely attributed to the NPC basket, a flexible subcomplex that protrudes into the nucleoplasm, providing a platform for regulatory factors.

**Figure 1.**
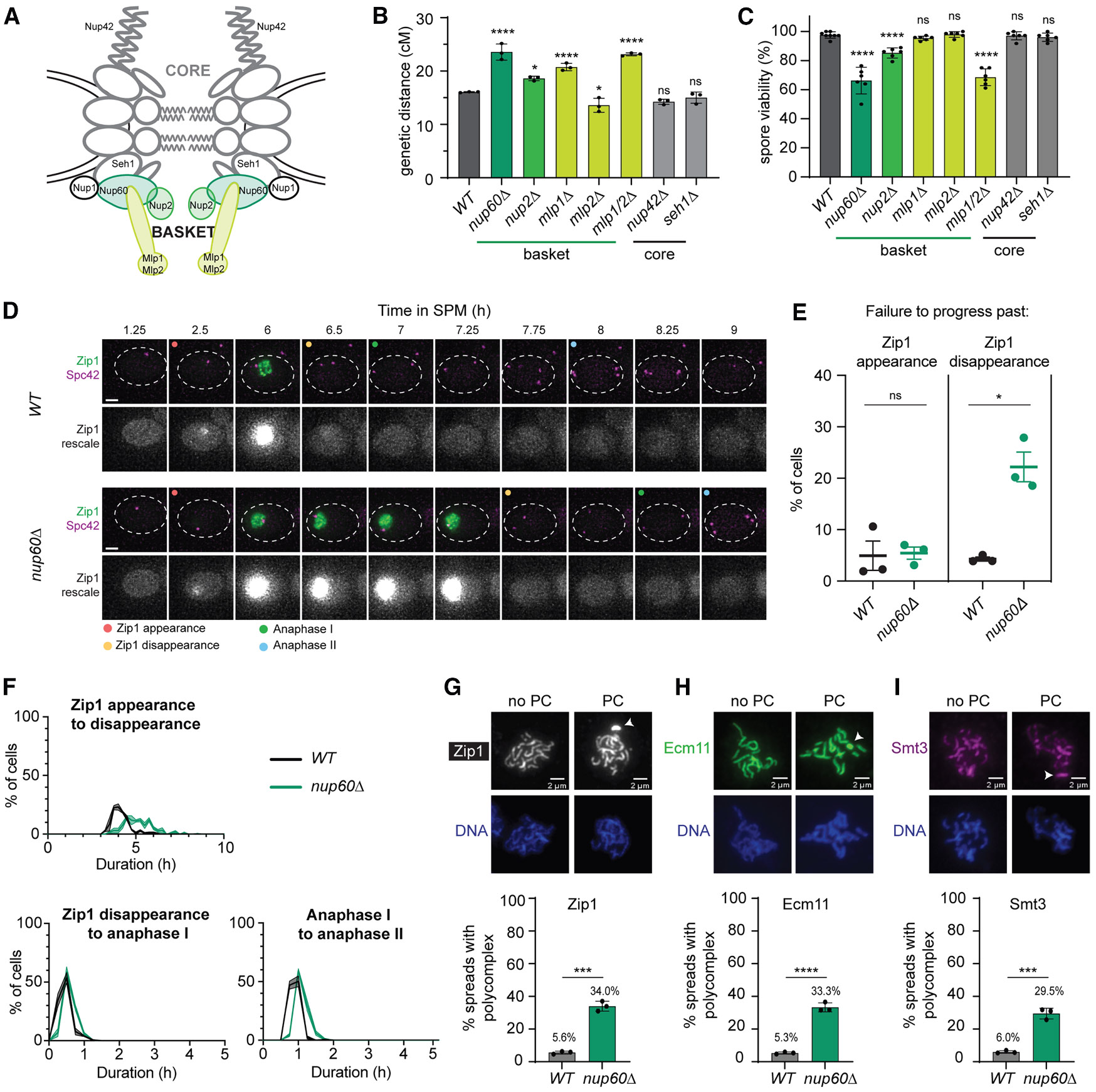
The NPC basket suppresses excessive crossing-over and supports chromosome synapsis (Related to Figure S1; Table S1, Videos S1 and S2) (A) Schematic representation of the *Saccharomyces cerevisiae* nuclear pore complex. The core and cytoplasmic subcomplexes are represented in gray. The nuclear basket is shown in green with names of individual basket proteins indicated. (B) Spore-autonomous fluorescence analysis of recombination frequency in strains with the indicated genotypes. The experiment was carried out in triplicates (*n* = 500 cells per genotype and replicate). Bar graph represents mean ± SD. (C) Spore viability assay of strains with the indicated genotypes. Six replicates were analyzed (*n* = 72 spores per genotype and replicate). Bar graph represents mean ± SD. (D) Montages from live-cell imaging depicting meiotic progression in strains with the indicated genotypes, monitored by Zip1^GFP^ and Spc42^mCherry^. Cells were imaged every 15 min after 1 h in SPM. Representative images are shown. Colored dots indicate time points scored as: Zip1 appearance (red), Zip1 disappearance (yellow), anaphase I (green), and anaphase II (blue). Scale bars, 2 μm. The full movies are available in Videos S1-S2. (E and F) Quantification of experiment in (D). (E) Percent of cells that fail to progress past Zip1 appearance or disappearance. The experiment was carried out in triplicates (*n* ≥ 100 cells per replicate). Only cells with initial Zip1 appearance were considered when calculating the percentage of cells in which Zip1 fails to disappear. The mean ± SE are depicted. (F) Duration of Zip1 presence (top), duration from Zip1 disappearance to anaphase I (bottom left), and duration from anaphase I to anaphase II (bottom right). Only cells which formed tetrads in the imaged time span were considered for analysis (*n* ≥ 40 tetrads per replicate). Lines and shaded ranges represent the mean ± SE of the triplicates. (G) Chromosome spreads from strains with the indicated genotypes co-stained for Zip1 and DAPI. Top: representative images with and without polycomplex (white arrowhead). Bottom: quantification of polycomplex formation on chromosome spreads. Only fully synapsed cells were scored. Experiment was performed in triplicates (*n* ≥ 100 cells per replicate). Bar graph represents mean ± SD. Scale bars, 2 μm. (H) As in (G), co-stained for SC protein Ecm11 and DAPI. (I) As in (G), co-stained for Smt3 (SUMO) protein and DAPI. Statistical testing: *p < 0.05; **p < 0.01; ***p < 0.005; ****p < 0.001; ns = non-significant by Dunnett’s multiple comparisons test (B and C) or by unpaired *t*-test (E, G, H, and I). *p* values in Table S1.

In budding yeast, the nuclear basket consists of Nup60, Nup1, Nup2, and the Mlp1-Mlp2 heterodimer, homologous to NUP153, NUP50, and TPR in metazoans (Figure 1A).^11-14^ Biochemical and structural work has revealed how Nup60 anchors the basket to the NPC core, providing a scaffold for Nup2 and the Mlp1-Mlp2 heterodimer via short linear motifs.^15-21^ In addition to tethering other basket proteins, Nup60 recruits non-nucleoporin factors, including the SUMO protease Ulp1, to the nuclear periphery.^22-24^ This spatial sequestration of Ulp1 is thought to shape substrate specificity^25,26^ and maintain SUMO homeostasis, a function conserved across species.^27^

The basket is the most plastic part of the NPC.^28^ Basket composition and localization change in response to cell cycle cues and post-translational modifications, thereby diversifying its functional repertoire.^29-34^ In vegetative cells, this plasticity allows the basket to act in processes such as DNA repair, chromatin organization, and transcriptional control.^6,8,35^ However, comparatively little is known about the functions of the basket in meiosis.

Several lines of evidence implicate basket components in yeast gametogenesis (sporulation) in yeast. Null mutants of *NUP2* or *NUP60* delay meiotic progression and have reduced spore viability, with phenotypes tied to Nup2’s chromatin-binding N-terminal region and Nup60’s role in anchoring Nup2 at the nuclear periphery.^21,36^ The basket is also highly dynamic during meiosis: Nup60 and Nup2 detach in a Polo-like kinase Cdc5-dependent manner from the NPC core in meiosis I and later reattach via an amphipathic helix in Nup60.^32^ In meiosis II, the entire basket detaches and is selectively inherited by spores.^37^ Despite these intriguing dynamics, how the nuclear basket regulates meiotic recombination and promotes gamete health remains unclear.

Meiotic recombination requires tightly regulated formation and repair of DNA double-strand breaks (DSBs) into crossovers to ensure accurate segregation of homologous chromosomes.^38^ Central to this regulation is the synaptonemal complex (SC), whose central element components, including Ecm11, undergo extensive SUMOylation.^39-41^ SUMO levels must be precisely balanced, since loss of SUMOylation destabilizes the SC and impairs recombination.^42-44^ Yet, how SUMO homeostasis is spatially and temporally regulated during meiosis has remained enigmatic.

Here, we uncover a mechanism by which the NPC basket promotes recombination fidelity. Most basket subunits are required to prevent excess crossovers and ensure gamete viability, with Nup60 emerging as the pivotal factor by tethering the SUMO protease Ulp1 to the NPC. Upon loss of Nup60, or when Ulp1 is delocalized from the nuclear periphery, Ecm11 SUMOylation is reduced, leading to SC destabilization, defective synapsis, and hyper-recombination. Importantly, engineered recruitment of Ulp1 to the NPC restores SC integrity and recombination control in basket mutants. Finally, we show that Cdc5 remodels the meiotic SUMO landscape at the prophase I-metaphase I transition by regulating the localization of a subset of Ulp1 and targeting the SUMO ligases Siz1 and Siz2. Together, these findings reveal how nuclear pore proteins, SUMO enzymes, and cell cycle kinases converge to control meiotic chromosome synapsis and crossing-over.

## RESULTS

### The NPC basket limits meiotic crossover formation and promotes gamete viability

To assess whether the NPC contributes to recombination control, we analyzed crossover frequencies at defined genomic intervals using the spore-autonomous fluorescence assay (Figure S1A).^45^ We compared deletion mutants of all non-essential basket nucleoporins (*NUP60*, *NUP2*, *MLP1*, and *MLP2*) alongside mutants lacking non-essential core components. For the latter, we selected Nup42, a cytoplasmic filament protein, and Seh1, a Y-complex member (Figure 1A).

Deletion of *NUP42* or *SEH1* did not alter recombination compared to wild type (Figure 1B, gray bars; Table S1), whereas deletion of basket components significantly increased crossover levels (Figure 1B, green bars; Table S1). The exception was *mlp2Δ*, which showed no increase alone, likely due to functional redundancy with Mlp1, as *mlp1Δmlp2Δ* double mutants did display elevated crossovers. Hyper-recombination in basket mutants correlated with reduced gamete viability, which dropped from 97.6 ± 2.2% (WT) to 66.2 ± 9.2% (*nup60Δ*), 85.2 ± 3.5% (*nup2Δ*), and 68.5 ± 5.7% (*mlp1Δmlp2Δ*). By contrast, spores from *mlp1Δ* and *mlp2Δ* single mutants, or from the non-basket mutants *nup42Δ* and *seh1Δ*, remained fully viable (Figure 1C; Table S1). These data suggest that meiotic recombination control is a basket-specific feature, not a general NPC property. Since Nup60 scaffolds the basket components^16,17,32,37^ and displayed the strongest phenotype, subsequent analyses focused on *nup60Δ*.

To determine whether excess crossovers arise through the canonical MutLγ (Mlh1-Mlh3) or Mus81-Mms4 pathways,^46-49^ we analyzed *nup60Δ* in combination with *mlh1Δ* or *mus81Δ*. Neither double mutant fully rescued the hyper-recombination phenotype, indicating that both pathways contribute to the increased number of crossovers (Figure S1B-S1C; Table S1). We note that Yen1, a third Holliday junction resolvase, acts late in meiosis as a backup to Mus81-Mms4.^48,50,51^ This likely explains the absence of a noticeable reduction in crossover frequency in the *mus81Δnup60Δ* double mutant compared with *nup60Δ* alone (Figure S1B; Table S1).

### Nup60 promotes SC integrity

Previous work reported delayed meiotic progression and reduced sporulation in *nup60Δ* without identifying the underlying cause.^21,36^ We therefore examined DNA replication, SC dynamics, and meiotic divisions. Flow cytometry analysis of DNA content confirmed that bulk DNA replication progressed with normal kinetics (Figure S1D). Live-cell imaging of Zip1^GFP^ indicated that SC assembly, assessed by the detection of structured Zip1^GFP^ signal, initiated at ~2.5 h after meiotic induction in wild-type and *nup60Δ* cells (Figure 1D, red dot; Figures S1E-S1G). However, 20% of *nup60Δ* cells completely failed to disassemble Zip1 and progress past prophase I (Figures 1E and S1E; Table S1). The remaining 80% maintained SC structures that persisted around one hour longer than wild type cells (in cells maturing into tetrads), delaying both meiotic divisions (Figures 1D and 1F, yellow dot, Videos S1 and S2). Once the meiotic divisions initiated, they proceeded with comparable kinetics in wild type and *nup60Δ* (Figures 1D and 1F, green and blue dots, Videos S1 and S2).

While most *nup60Δ* cells assembled and eventually disassembled the SC, chromosome spread analysis revealed that at least 34% of *nup60Δ* cells formed Zip1 assemblies known as “polycomplexes”, compared to only 5% in wild type, indicating compromised SC dynamics (Figure 1G). Similar defects were observed for the SC central element Ecm11 and SUMO (Smt3), which decorates the SC (Figures 1H and 1I).^40,42^ Thus, Nup60 promotes efficient SC assembly/maintenance, and defects in these dynamics may contribute to the hyper-recombination phenotype in NPC basket mutants.

### Mislocalization of Ulp1 underlies the hyper-recombination phenotype in *nup60Δ* cells

**Figure 2.**
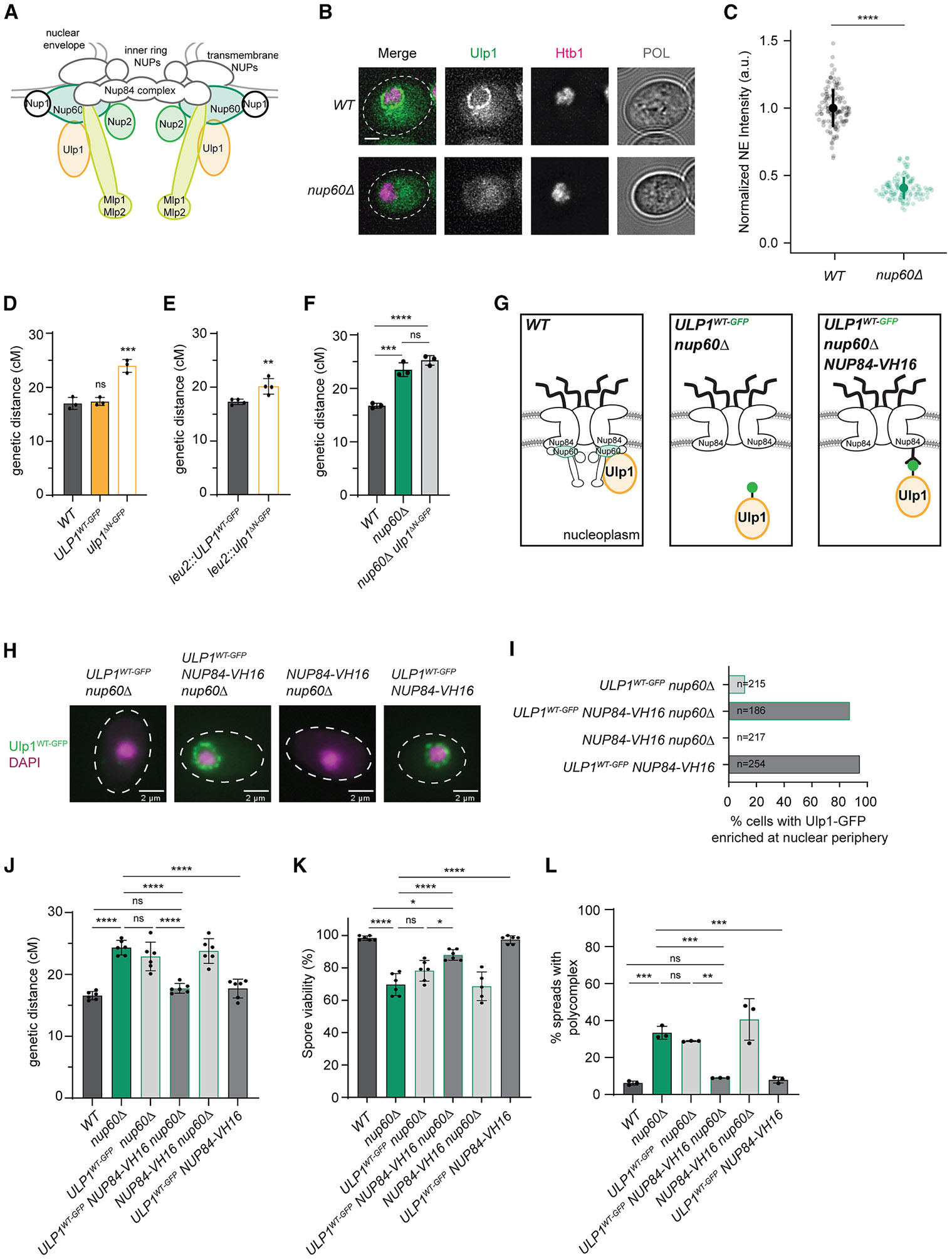
Tethering of Ulp1 to the nuclear periphery via Nup60 prevents excessive crossing-over (Related to Table S1) (A) Schematic representation of Ulp1 localization to the *S. cerevisiae* nuclear basket based on Singh et al. 2024.^17^ (B) Live-cell imaging of strains with the indicated genotypes using Ulp1^WT-GFP^ and Htb1^mCherry^. Cells were imaged after ~5 h in SPM. Scale bars, 2 μm. (C) Quantification of Ulp1^WT-GFP^ intensity at the nuclear envelope for (B). Nuclear envelope (NE) intensity for each cell was normalized to the mean nuclear envelope intensity observed in *WT* (*n* ≥ 100 cells per genotype). (D) Spore-autonomous fluorescence analysis of recombination frequency in strains with the indicated genotypes. The experiment was carried out in triplicates (*n* = 500 cells per genotype and replicate). Bar graph represents mean ± SD. (E) As in (D) for strains with the indicated genotypes. (F) As in (D) for strains with the indicated genotypes. (G) Schematic representation of the genetic setup used in Figures 2H-2L. Ulp1 (orange) is internally tagged with GFP. Nup60 is depicted in green. Left: *WT* scenario; middle: in *nup60Δ background* Ulp1^WT-GFP^ is mis-localized to the nucleoplasm; right: *nup60Δ NUP84-VH16* background, with Nup84 tagged by a VH16 nanobody, anchoring Ulp1^WT-GFP^ to the nuclear periphery. (H) Representative images of indicated genotypes. Ulp1^WT-GFP^ is visualized using the GFP tag. DNA is visualized with DAPI. Scale bars, 2 μm. (I) Quantification of Nup84-VH16/Ulp1^WT-GFP^ tether efficiency shown in (H). Cells with Ulp1^WT-GFP^ at the nuclear periphery were scored. Number of quantified cells *(n)* is indicated in graph. (J) As in (D) for strains with the indicated genotypes. (K) Spore viability assay of strains with the indicated genotypes. Six replicates were analyzed (*n* = 72 spores per genotype and replicate). Bar graph represents mean ± SD. (L) Quantification of polycomplex formation in cells of indicated genotypes. Only fully synapsed cells were scored. Experiment was performed in triplicates (*n* ≥ 100 cells per replicate). Bar graph represents mean ± SD. Statistical testing: *p < 0.05; **p < 0.01; ***p < 0.005; ****p < 0.001; ns = non-significant by Wilcoxon rank-sum test (C), by Dunnett’s multiple comparisons test (D), by unpaired *t*-test (E) or by Tukey’s multiple comparisons test (F, J, K, and L). *p* values in Table S1.

A prime effector through which the basket might influence recombination is Ulp1, an essential SUMO protease tethered to the NPC through Nup60, Mlp1, Mlp2, and Nup84^24,52^ (Figure 2A). Live imaging confirmed that, similar to vegetative cells,^22^ Ulp1^WT-GFP^ levels at the nuclear periphery were greatly reduced in meiotic *nup60Δ* cells (Figures 2B and 2C; Table S1).

To test whether Ulp1 delocalization was sufficient to cause excess recombination, we examined cells expressing a truncated allele of *ULP1* (*ulp1^ΔN-GFP^*) that cannot bind the NPC.^53^ These cells displayed elevated crossover frequency (23.9 ± 1.2 cM) relative to wild-type (17.6 ± 1.4 cM) or *ULP1^WT-GFP^* controls (17.3 ± 0.7 cM) (Figure 2D; Table S1). Next, to determine whether increased crossing-over resulted from loss of Ulp1 function at the nuclear periphery or from its increased abundance in the nucleoplasm, we generated strains carrying either *ulp1^ΔN-GFP^* or *ULP1^WT-GFP^* as a transgene integrated at an ectopic site, while maintaining the wild-type *ULP1* allele at its endogenous site. Remarkably, *ulp1^ΔN-GFP^*, but not *ULP1^WT-GFP^*, acted in a dominant manner (Figures 2D and 2E; Table S1). Therefore, elevated crossover frequency results from increased Ulp1 activity in the nucleoplasm rather than reduced Ulp1 activity at the nuclear periphery.

To further investigate whether the recombination phenotype of *nup60Δ* arises from Ulp1 mislocalization, we combined *nup60Δ* with *ulp1^ΔN-GFP^*. Rather than the additive increase expected if the two acted in parallel pathways, crossover frequencies in the double mutant were indistinguishable from either single mutant (Figure 2F; Table S1). This genetic non-additivity places Nup60 and Ulp1 in the same pathway, consistent with a role for Nup60 functioning as an NPC tether for Ulp1.

### Engineered tethering of Ulp1 restores recombination control

If untethered Ulp1 drives excessive crossovers in *nup60Δ* cells, restoring its NPC localization should restore crossover formation to wild-type levels. To test this prediction, we fused a GFP nanobody (VH16)^54^ to the core nucleoporin Nup84, capturing Ulp1^WT-GFP^ at the NPC independently of Nup60 (Figures 2G-2I). Remarkably, tethering Ulp1 to Nup84 reduced crossover frequencies in the *nup60Δ* background to wild-type levels, improved spore viability, and reduced polycomplex formation (Figures 2J-2L; Table S1). These data establish that Nup60 restrains recombination and preserves SC integrity by anchoring Ulp1 to the NPC, thereby preventing its deleterious activity in the nucleoplasm.

### Nucleoplasmic Ulp1 disrupts Ecm11 SUMOylation

**Figure 3.**
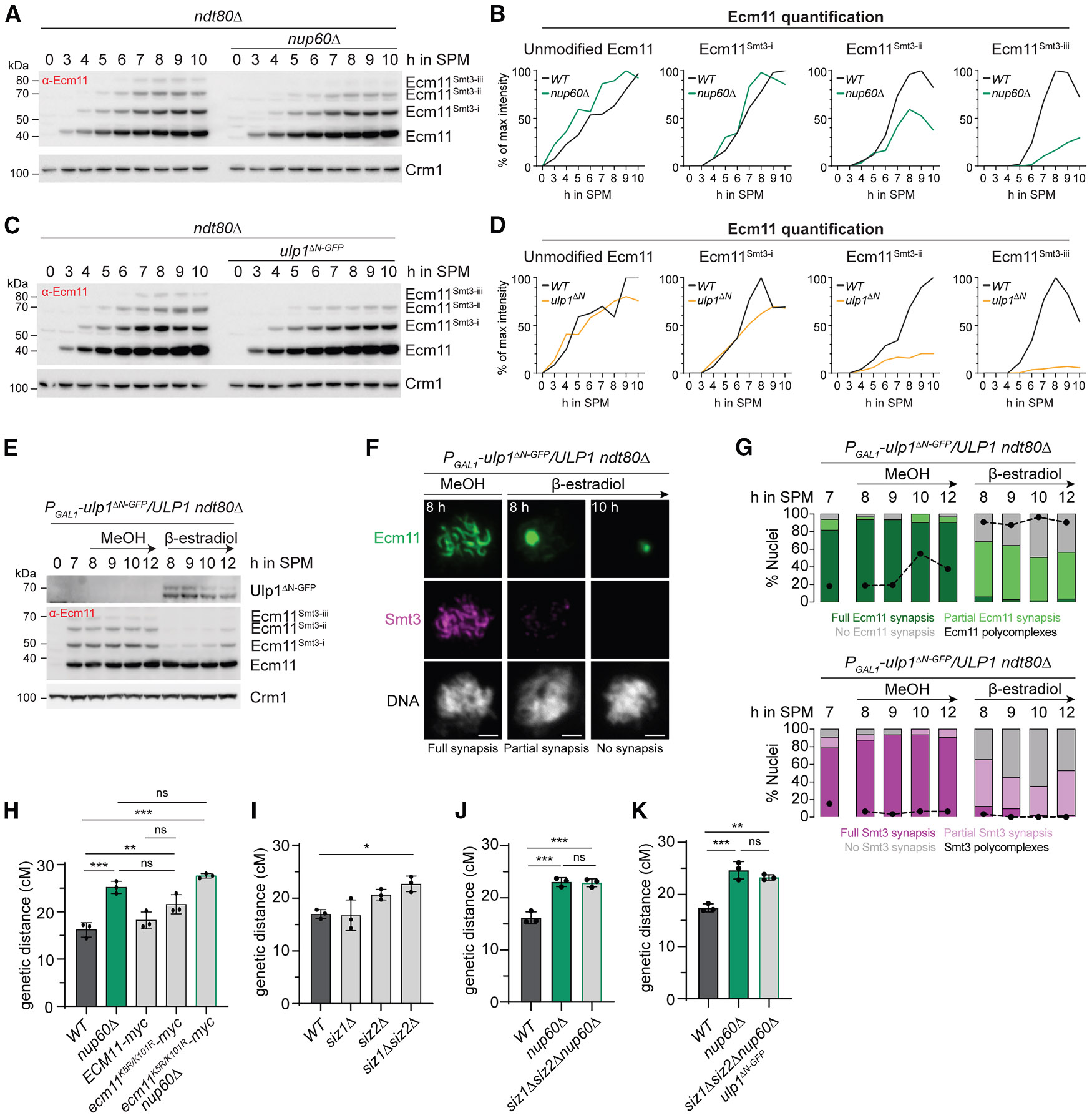
Anchoring the SUMO protease Ulp1 to the NPC prevents Ecm11 deSUMOylation (Related to Figure S2; Table S1) (A) Westen blot analysis of strains with the indicated genotypes in meiosis. Samples were collected at specified times in SPM. Anti-Ecm11 antibody was used to detect Ecm11 and its modified versions. Ecm11^Smt3–i-iii^ is used to indicate different modified versions of Ecm11 protein. Crm1 was used as normalization control. (B) Quantification of (A), separated by different SUMO-modified versions of Ecm11. Crm1 signal was used for normalization of Ecm11 signal. (C) Western blot analysis of strains with the indicated genotypes in meiosis as in (A). (D) Quantification of (C), as in (B). (E) Western blot analysis of Ecm11 and Ulp1^ΔN-GFP^ in extracts from cells with the indicated genotype at specified times in SPM. Ulp1^ΔN-GFP^ was induced by addition of 2 μM β-estradiol (or MeOH as control) at 7 h in SPM. Crm1 served as normalization control. (F) Representative images of meiotic chromosome spreads of cells with the indicated genotypes at specified times in SPM from the experiment in (E), immunostained for Ecm11 (green) and Smt3 (magenta). DNA was visualized using DAPI (gray). Scale bars, 2 μm. (G) Quantification of Ecm11 and Smt3 synapsis from (F), showing fully synapsed (dark green/magenta), partially synapsed (bright green/lilac), and non-synapsed SCs (gray). Black dotted line indicates fraction of nuclei with a polycomplex. 50 nuclei were analyzed per time point. (H) Spore-autonomous fluorescence analysis of recombination frequency in strains with the indicated genotypes. The experiment was carried out in triplicates (*n* = 500 cells per genotype and replicate). Bar graph represents mean ± SD. (I–K) As in (H) for strains with the indicated genotypes. Statistical testing: *p < 0.05; **p < 0.01; ***p < 0.005; ns = non-significant by Tukey’s multiple comparisons test (H, J, and K) or by Dunnett’s multiple comparisons test (I). *p* values in Table S1.

We next asked which targets of mislocalized Ulp1 were responsible for the phenotypes observed in the *nup60Δ* mutant. Ecm11, a central SC component, is heavily SUMOylated and required for crossover regulation.^40^ Immunoblotting revealed multiple SUMO-modified Ecm11 species in prophase-I-arrested cells (*ndt80Δ*), as previously reported^40^ (Figures 3A and S2A). These lower-mobility forms were reduced in *nup60Δ* and *ulp1^ΔN-GFP^* mutants (Figures 3A-3D), consistent with aberrant SUMOylation patterns caused by mislocalized Ulp1 (Figures S2B-S2E).

To test whether delocalized Ulp1 could remove SUMO from pre-existing SC structures, we induced Ulp1^ΔN-GFP^ expression in prophase-I-arrested cells.^44^ Briefly, *ulp1^ΔN-GFP^* (or *ULP1^WT-GFP^*) was placed under the control of a β-estradiol-inducible promoter, enabling temporally controlled expression of *ulp1^ΔN-GFP^* (or *ULP1^WT-GFP^*).^55^ As anticipated, SUMOylated Ecm11 species were depleted upon Ulp1^ΔN-GFP^ expression, accompanied by loss of chromosome-associated Ecm11 and a concomitant increase in Ecm11 polycomplex formation (Figures 3E-3G). These effects were partially reversed as Ulp1^ΔN-GFP^ expression declined over time, allowing SUMOylation to be slowly re-established. We additionally noted that ectopic Ulp1^ΔN-GFP^ expression resulted in a more severe reduction of Ecm11 SUMOylation than observed in *nup60Δ* mutants. This is likely explained by overall lower levels of Ulp1 present in the *nup60Δ* background (Figure S2F) and hints at a dosage-dependent effect. Inducing wild-type Ulp1^WT-GFP^ did not noticeably affect Ecm11 SUMOylation or chromosome synapsis (Figures S2G and S2H). Thus, nucleoplasmic Ulp1 targets SUMOylated Ecm11, thereby destabilizing the SC.

### Perturbing Ecm11 SUMOylation phenocopies basket and Ulp1 mutants

To determine whether altered SUMOylation of Ecm11 accounts for the recombination phenotype, we analyzed non-SUMOylatable Ecm11 mutants. As reported, *ecm11^K5R/K101R^* mutants displayed elevated crossover frequency.^40^ Importantly, this was non-additive with *nup60Δ* (Figure 3H; Table S1), implying that regulation of Ecm11 SUMOylation is one of the mechanisms by which Nup60 modulates recombination.

Since Ecm11 SUMOylation is catalyzed primarily by the SUMO ligases Siz1 and Siz2,^41^ we tested whether their loss affected crossing-over. Indeed, *siz1Δsiz2Δ* double mutants showed elevated crossover levels (Figure 3I; Table S1). Notably, *siz1Δsiz2Δ* crossover levels were not additive with *nup60Δ* or in a *siz1Δsiz2Δnup60Δulp1*^*ΔN–GFP*^ quadruple mutant background (Figures 3J and 3K; Table S1). These epistasis analyses indicate that Nup60, Ulp1, and Siz1/2 act in a shared pathway to safeguard Ecm11 SUMOylation, thereby stabilizing the SC and limiting crossover recombination.

### Cdc5-mediated release of Ulp1 from the nuclear periphery during meiosis I

We previously reported the transient, Cdc5-mediated, detachment of Nup60 from the nuclear envelope in meiosis I.^32^ Since Ulp1 localization relies on Nup60 (Figures 2B and 2C), we hypothesized that Ulp1 might undergo a similar regulated release. To test this, we employed live-cell imaging of Ulp1^WT-GFP^ in strains with Htb1^mCherry^. Ulp1^WT-GFP^ exhibited a subtle but significant reduction in nuclear periphery localization during anaphase I when compared to the core nucleoporin Pom34 (Figures S3A and S3B; Table S1, Video S3).^32^ Ulp1 detachment occurred with similar timing to Nup60 but was less pronounced, possibly reflecting residual interactions with alternative NPC anchors.^56-58^

**Figure 4.**
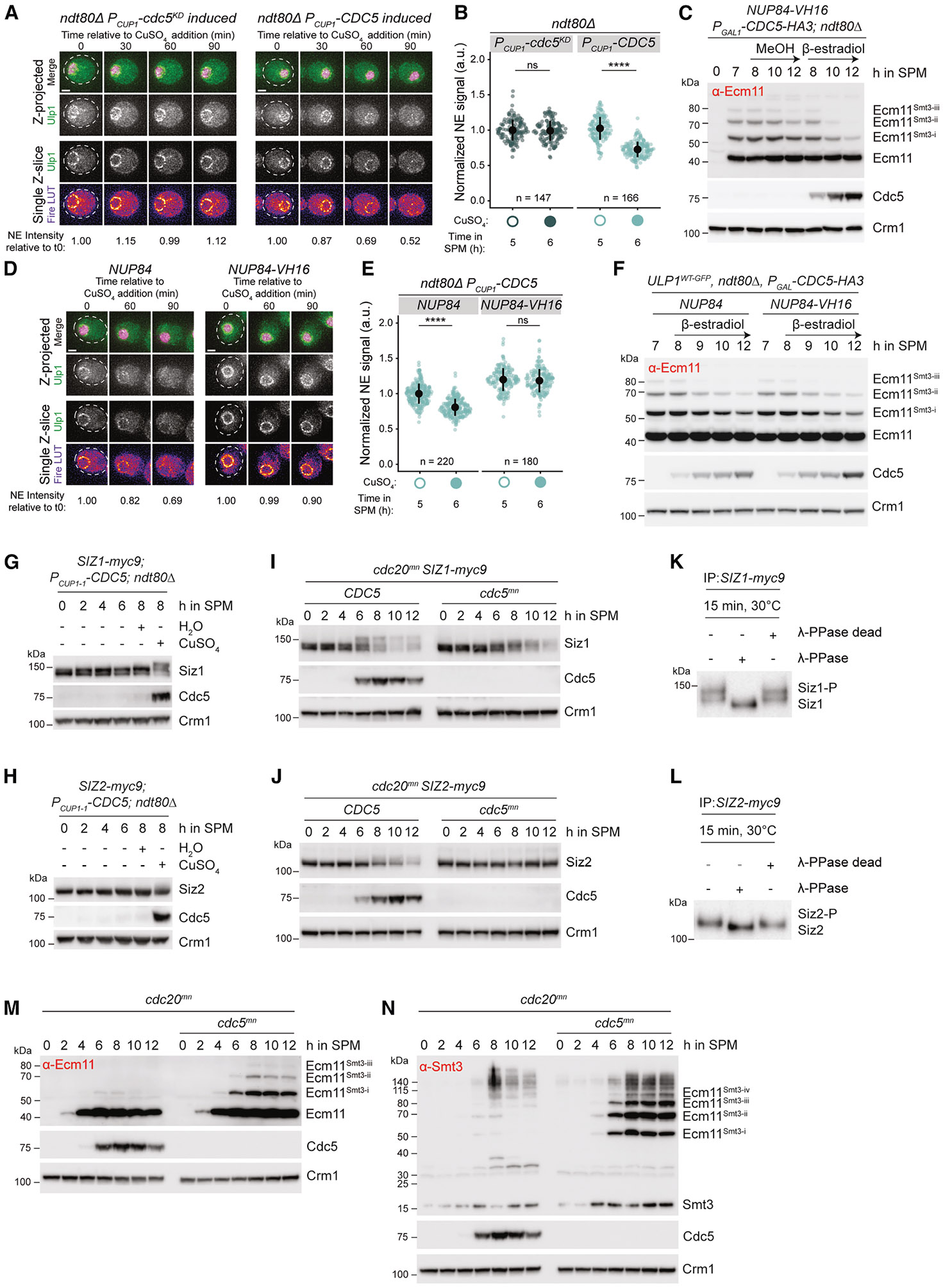
Cdc5 regulates SUMOylation by coordinately targeting SUMO ligases and SUMO proteases (Related to Figures S3 and S4; Table S1, Videos S4, S5, and S6) (A and B) Montages from live-cell imaging of strains with the indicated genotypes using Ulp1^WT-GFP^ and Htb1^mCherry^ fluorescent tags. *P_CUP1_-CDC5-3xFLAG-10xHis* expression was induced at 5 h in SPM with 50 μM CuSO_4_. Cells were imaged in 15 min intervals post-induction. Representative images are shown. Scale bars, 2 μm. The full movies can be found in Videos S4 and S5. KD: kinase dead (B) Quantification of Ulp1 detachment in (A). Nuclear envelope (NE) intensity values were normalized to the average nuclear envelope intensity for *P_CUP1_-cdc5^KD^-3xFLAG-10xHis* cells at the pre-induction time point (5 h in SPM). Post-induction (6 h in SPM) values were compared to pre-induction (5 h in SPM) values for each treatment. Sample sizes *(n)* are the number of cells quantified for each treatment regimen. (C) Western blot analysis of strain with the indicated genotype. Inducing agent (β-estradiol, 2 μM) or vehicle (MeOH) was added at 7 h in SPM. Samples were collected at specified times in SPM. Anti-HA antibody was used to detect Cdc5. Anti-Ecm11 antibody was used to detect Ecm11 and its modified forms. Crm1 served as normalization control. (D) Montages from live-cell imaging of strains with the indicated genotypes as in (A). Representative images are shown. Scale bars, 2 μm. The full movie can be found in Video S6. (E) Quantification of Ulp1 detachment in (D). Nuclear envelope intensity values were normalized to the average nuclear envelope intensity for *NUP84* cells at the pre-induction time point (5 h in SPM). Values were compared as in (B). Sample sizes (*n*) are the number of cells quantified for each treatment regimen. (F) Western blot analysis of strains with the indicated genotypes in meiosis. *P_GAL1_-CDC5-3HA* expression was induced by addition of 2 μM β-estradiol at 7 h in SPM. Samples were collected at specified times and proteins were detected as in (C). (G) Western blot analysis of Siz1 protein in strain of the indicated genotype. Cdc5 expression was induced at 7 h in SPM by addition of 50 μM CuSO_4_. Samples were collected at specified times in SPM. Siz1 protein was detected using an anti-Myc antibody. Cdc5 was detected with an anti-Cdc5 antibody. Crm1 served as normalization control. (H) Western blot analysis as in (G) for Siz2 protein. (I) Western blot analysis of Siz1 protein in strains of the indicated genotypes. Samples were collected at specified times in SPM. Siz1 protein was detected using an anti-Myc antibody. Cdc5 was detected with an anti-Cdc5 antibody and indicates progression in meiosis. Crm1 served as normalization control. (J) Western blot analysis of Siz2 protein in strains of the indicated genotypes as in (I). (K) Siz1-myc9 tagged protein was immuno-affinity purified from metaphase I cultures (*cdc20^mn^* background, 10 h in SPM). Proteins were treated with native or inactivated λ-phosphatase (λ-PPase), as indicated, and analyzed by western blotting. (L) As in (K) for Siz2-myc9 tagged protein. (M) Western blot analysis of Ecm11 protein in strains with the indicated genotypes. The same samples as in (I) were used. Anti-Ecm11 antibody was used to detect Ecm11 and its modified forms. Crm1 served as normalization control. (N) Western blot analysis of Smt3 protein in strains of the indicated genotypes. The same samples as in (I) were used. Smt3 antibody was used to detect SUMO. Crm1 served as normalization control. Statistical testing: ****p < 0.001; ns = non-significant by Wilcoxon singed-rank test (B and E). *p* values in Table S1.

To establish whether this release was dependent on Cdc5, we monitored Ulp1^WT-GFP^ localization in *ndt80Δ* cells expressing an inducible *CDC5* allele. In prophase I (5 h in sporulation medium [SPM]), Ulp1^WT-GFP^ localized to the nuclear periphery. Upon induction of *CDC5^WT^* (6 h in SPM), Ulp1^WT-GFP^ delocalized from the nuclear periphery, whereas it remained at the nuclear periphery when a kinase-dead allele (*cdc5^KD^*)^59^ was expressed (Figures 4A and 4B; Table S1; Videos S4 and S5). Notably, Ulp1 detachment was more robust in this context than in wild-type meiosis, likely due to higher levels and/or longer exposure to Cdc5 activity (compare Figure 4B to Figure S3B). To further investigate the mechanism and function of Ulp1 detachment, we used the same system in follow-up experiments.

Since Cdc5-dependent phosphorylation of Nup60 promotes its detachment from the NPC,^32^ we sought to determine whether the same phosphorylation sites were required for Ulp1 release. Interestingly, Ulp1^WT-GFP^ still detached from the nuclear periphery upon *CDC5^WT^* induction in a *nup60^9A^* background (a phosphorylation-resistant mutant preventing Nup60 detachment^32^), indicating that Nup60 retention at the nuclear periphery is not sufficient to maintain Ulp1 there upon elevated Cdc5 activity (Figures S3C and S3D; Table S1). Therefore, additional mechanisms, besides Nup60 phosphorylation, must be in place to trigger Ulp1 detachment. This suggests that cells actively promote Ulp1 release in meiosis I, rather than Ulp1 being passively redistributed because of Nup60 detachment.

Consistent with a broader regulatory role for Cdc5, ectopic expression of Cdc5 in pachytene-arrested cells (*ndt80Δ*, 7 h in SPM) triggered loss of SUMOylated Ecm11 (Figure 4C). To test whether Ulp1 release from the NPC was required for this process, we combined the Ulp1-NPC tether (Figure 2G) with inducible *CDC5*. Live-cell imaging confirmed that the Nup84-VH16 tether efficiently prevented Ulp1^WT-GFP^ detachment, even upon ectopic Cdc5 expression (Figures 4D and 4E; Table S1, Video S6). However, immunoblotting revealed that the loss of SUMOylated Ecm11 occurred with similar kinetics and extent, regardless of whether Ulp1 was tethered or released (Figures 4F, S3E-S3J). Thus, although Ulp1 is transiently released from the NPC during meiosis and can deSUMOylate Ecm11, its release is not strictly required for Ecm11 deSUMOylation when Cdc5 activity is elevated. Furthermore, meiotic progression was unaffected when Ulp1 was constitutively tethered at the nuclear periphery (Figures S3K and S3L).

### Cdc5 kinase regulates SUMO ligases Siz1 and Siz2

Given the aforementioned findings, we reasoned that Cdc5 might also regulate Ecm11 SUMOylation through additional components of the SUMO machinery. Ulp2—the second SUMO protease in yeast and a Cdc5 target in mitotic cells^60,61^—is required for meiotic progression past prophase I.^41^ We therefore analyzed whether potential post-translational modifications on Ulp2 during meiosis depend on Cdc5. To this end, we monitored Ulp2 in metaphase-arrested cells (*cdc20^mn^*) in presence or absence of Cdc5 (*cdc5^mn^*) using the *CLB2* promoter to deplete the respective proteins specifically in meiosis.^62^ Ulp2 displayed a prominent electrophoretic mobility shift ~2 h after transfer to SPM, coinciding with pre-meiotic S phase or early prophase I (Figures S4A and S4B). This shift correlates temporally with phosphorylation events detected in our previous meiotic phosphoproteomic analyses.^63^ However, it did not correlate with Cdc5 expression, which accumulated several hours later and was also observed in *cdc5^mn^* cells. These findings suggest that Ulp2 modification during meiosis occurs largely independently of Cdc5.

Among other candidate factors, the SUMO ligases Siz1 and Siz2 stood out for three reasons: (1) we previously identified Siz1 as a phosphorylation target of Cdc5^32^; (2) deletion of *SIZ2* or co-deletion of *SIZ1* and *SIZ2* increased crossover frequencies in a manner non-additive with *nup60Δ* (Figures 3J and 3K), implying a shared pathway; (3) Siz1 and Siz2 are the E3 ligases responsible for Ecm11 SUMOylation.^41^ We therefore hypothesized that Cdc5 may modify Siz1, Siz2, or both to influence their function.

To test this, we analyzed Siz1 and Siz2 upon ectopic induction of Cdc5 expression in pachytene-arrested cells. Notably, Cdc5 accumulation was sufficient to induce electrophoretic mobility shifts in both Siz1 and Siz2 (Figures 4G and 4H). Moreover, Cdc5 was required for these modifications in metaphase-I-arrested cells (*cdc20^mn^* vs. *cdc20^mn^cdc5^mn^*), indicating that the full mobility shift of Siz1 and Siz2 depends on Cdc5 accumulation (Figures 4I and 4J). Finally, phosphatase treatment abolished the Cdc5-dependent mobility shifts of Siz1 and Siz2, confirming that they reflect phosphorylation (Figures 4K and 4L).^32^

Because Siz1 and Siz2 are the principal E3 ligases for Ecm11,^41^ we next asked whether Cdc5-dependent modification of these enzymes correlated with changes in Ecm11 SUMOylation. Strikingly, SUMOylated forms of Ecm11 accumulated only transiently and decreased coincidently with increased Cdc5 expression, whereas they accumulated continuously in the absence of Cdc5 (Figure 4M). Moreover, in the absence of Cdc5, Ecm11 persisted on chromosomes into metaphase I (Figures S4C and S4D). This temporal relationship suggests that Cdc5 not only phosphorylates Siz1/2 but also promotes turnover of SUMOylated Ecm11.

### Cdc5 kinase remodels the SUMO proteome at the meiosis I onset

Finally, we asked whether Cdc5 regulates SUMOylation more broadly. To address this, we examined global SUMO conjugation patterns in extracts from *cdc20^mn^* and *cdc20^mn^cdc5^mn^* strains. In cells expressing Cdc5 (*cdc20^mn^*), SUMO-modified proteins accumulated only transiently during the metaphase I arrest, whereas in the absence of Cdc5 (*cdc20^mn^cdc5^mn^*), cells displayed persistent accumulation of a broad range of SUMO conjugates, including high-molecular weight species (Figure 4N). Together with our findings on Ulp1 relocalization and Siz1/Siz2 phosphorylation, these results suggest that Cdc5 helps reshape the meiotic SUMO machinery by coordinating Ulp1 redistribution with phosphorylation-dependent regulation of Siz1 and Siz2.

## DISCUSSION

**Figure 5.**
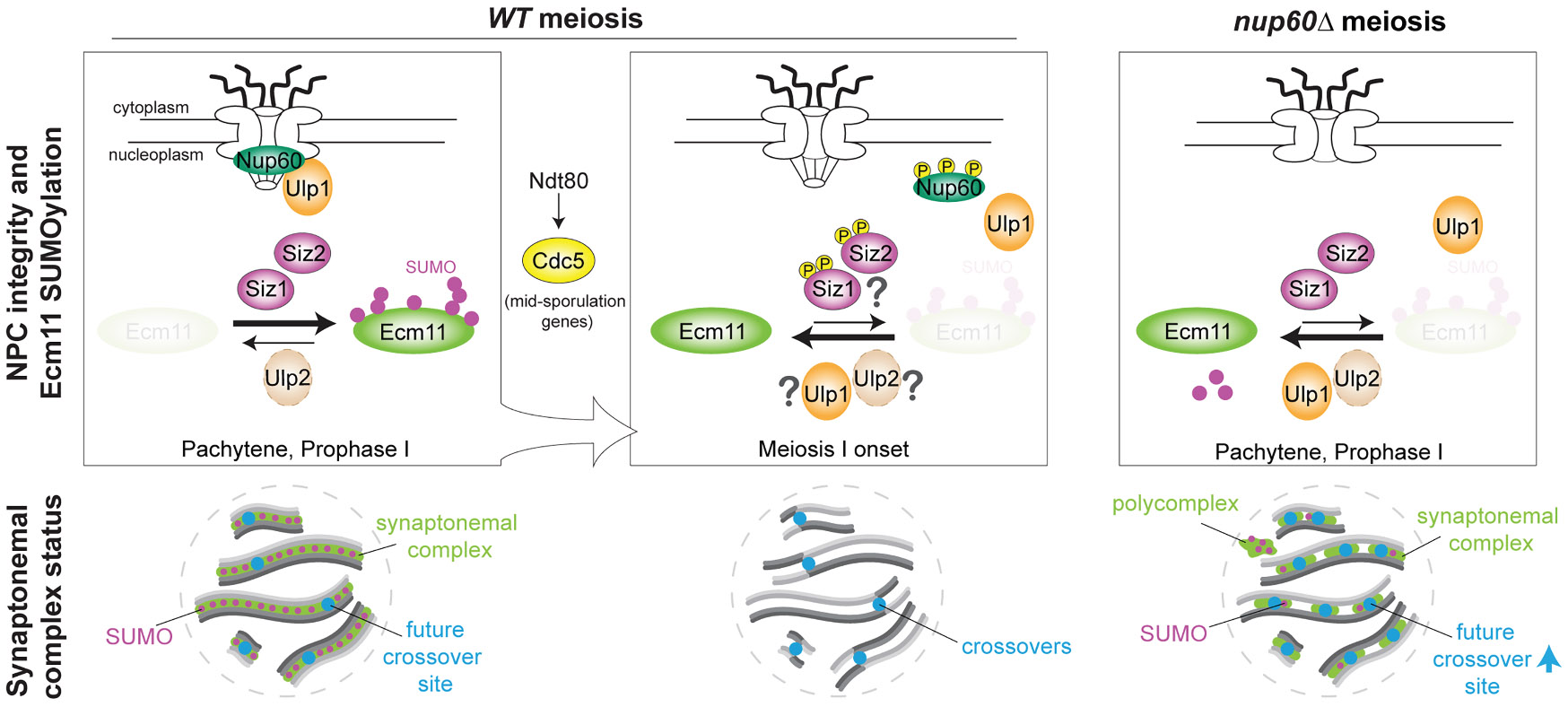
Working model During pachytene of meiotic prophase I (left), Ulp1 is anchored at the nuclear basket through Nup60, restricting its activity to the nuclear periphery. Under these conditions, the synaptonemal complex (SC) central element protein Ecm11 is highly SUMOylated, promoting stable SC assembly and proper designation of crossover sites. At the prophase I-metaphase I transition (middle), Cdc5 expression remodels the SUMO landscape by promoting partial release of Nup60 and Ulp1 from the NPC and phosphorylation of the SUMO ligases Siz1 and Siz2. This coordinated regulation reduces Ecm11 SUMOylation and potentially facilitates SC disassembly and progression into meiotic divisions. Future work is required to elucidate the specific roles and regulation of Siz1, Siz2, and Ulp2, in this process. For simplicity, the pool of Ulp1 that remains NPC anchored at the onset of meiosis I is not depicted. In the absence of Nup60 (right), Ulp1 is constitutively delocalized to the nucleoplasm. This leads to premature deSUMOylation of Ecm11 during prophase I, destabilization of the SC, polycomplex formation, and elevated crossover formation, ultimately compromising gamete viability.

We identify the NPC basket as a regulator of meiotic SUMO homeostasis and crossover control. Nup60 anchors the SUMO protease Ulp1 at the nuclear envelope, enabling appropriate SUMOylation of the SC component Ecm11 and stable synapsis. Untimely nucleoplasmic accumulation of Ulp1 in *nup60Δ* mutants disrupts SC integrity and leads to hyper-recombination-defects that can be fully rescued by re-anchoring Ulp1 to the nuclear periphery (Figure 5). Thus, NPCs function beyond nucleocytoplasmic transport to act as spatial organizers of signaling enzymes during meiosis, echoing findings from vegetative cells where basket nucleoporins coordinate DNA repair, transcription, and chromatin regulation through selective recruitment of regulatory factors.^3,4,6-10,35^

### The NPC-SUMO axis coordinates SC stability, crossover control, and gamete viability

In mutants that fail to anchor Ulp1 to the NPC basket, we observed that premature deSUMOylation of Ecm11 coincided with the accumulation of polycomplexes and prolonged prophase I progression (Figures 1 and 3). We propose that impaired SC dynamics represent a direct consequence of defective Ecm11 SUMOylation. By contrast, the prophase I delay/arrest is likely an indirect consequence of defective SC assembly or maintenance, which were reported to be required for the suppression of *de novo* DSB formation.^44,64-66^ This model is consistent with previous studies reporting elevated DSB levels in both SC central element mutants and *nup2Δ* cells.^27,36,64,65^ We hypothesize that defects in Ecm11 SUMOylation disrupt the SC-mediated feedback inhibition that normally limits DSB formation after cells accumulate crossover-marked recombination intermediates. The resulting continuous DSB accumulation leads to excessive recombination and unregulated crossover events. The prolonged persistence of DSB repair intermediates likely activates the DNA damage response, thereby delaying meiotic progression.^67^

Importantly, although the similarity between *ecm11^K5R/K101R^* and *ulp1^ΔN-GFP^* mutants highlights Ecm11 as a critical target, the fact that the *nup60Δ* or *ulp1^ΔN-GFP^* mutant phenotype is more severe suggests that additional substrates are likely involved (Figure 3). Other SC components or recombination factors may undergo premature deSUMOylation, further amplifying the hyper-recombination phenotype. Identifying these additional SUMO targets and determining whether their misregulation directly perturbs recombination or influences other aspects of meiosis will be essential for a more comprehensive understanding of how SUMO dynamics coordinate meiotic chromosome behavior and safeguard meiotic progression.

Although we identify Ulp1 tethering as a key role of the nuclear basket during meiosis, additional functions are likely. Tethering Ulp1 to the nuclear periphery in a *nup60Δ* mutant only partially restored spore viability (Figure 2K), which may reflect incomplete rescue by constitutive tethering or roles of Nup60 that extend beyond Ulp1 anchoring. Supporting the latter notion, deletion of *NUP2* led to moderately increased recombination and reduced spore viability, even though Nup2 has been reported as dispensable for Ulp1 localization in vegetative cells.^68^ Whether this also holds true in meiosis remains to be determined, but Nup2 has additionally been proposed to interact directly with chromosomes,^36^ a process which is linked to Nup2-Nup60 interaction.^21^ Further, meiotic regulation of Ulp1 tethering appears to be independent of previously described nuclear basket remodeling events during meiosis I and meiosis II (Figure S3), suggesting that nuclear basket dynamics may be involved in additional processes. Thus, the cellular defects observed in NPC basket mutants likely represent a multi-layered phenotype.

### Cdc5 rewires the SUMO proteome at the prophase I-metaphase I transition

Our work demonstrates that Polo-like kinase Cdc5 orchestrates a major remodeling of the meiotic SUMO landscape. Cdc5 simultaneously promotes the transient release of Ulp1 from the nuclear periphery and drives phosphorylation of the SUMO ligases Siz1 and Siz2 (Figure 4).^32^ This dual regulation likely provides robust and complementary control: mobilization of Ulp1 enhances deSUMOylation capacity, while phosphorylation of Siz1/2 correlates with reduced accumulation of SUMOylated proteins. Importantly, however, several aspects of this regulation remain unresolved. Future work will be needed to determine whether Cdc5 directly phosphorylates Siz1/2, whether these modifications alter ligase function, and whether other SUMO ligases or cofactors are also subject to Cdc5 regulation.

### Broader impact

Our work provides a mechanistic explanation for the recombination and sporulation defects observed in nuclear basket mutants, directly linking them to misregulation of protein SUMOylation. This dysregulation ultimately compromises gamete viability. Given that Ulp1^SENP1-2^ tethering to NPCs and Siz1/2^PIAS1-4^-mediated SUMOylation are conserved in other organisms,^27^ analogous mechanisms may operate in metazoan gametogenesis, where defects in SC dynamics and crossover control contribute to aneuploidy and infertility.

### Limitations of the study

While our findings establish a central role for Nup60-dependent Ulp1 tethering in regulating SC SUMOylation and crossover control, the following limitations should be considered. First, although Ulp1 mislocalization is sufficient to disrupt Ecm11 SUMOylation during pachytene, our experiments do not exclude the possibility that NPC-associated Ulp1 contributes to Ecm11 deSUMOylation at later stages of meiosis, in particular upon SC disassembly as cells enter meiosis I. Second, although Cdc5-dependent phosphorylation of the SUMO ligases Siz1 and Siz2 correlates with reduced accumulation of SUMOylated Ecm11, the functional consequences of these modifications remain unclear. Finally, while our data suggest that Ulp2 modification during meiosis occurs largely independently of Cdc5, a Cdc5-dependent role for Ulp2 in meiotic SUMO regulation cannot be excluded.

## STAR★METHODS

### EXPERIMENTAL MODEL AND STUDY PARTICIPANT

#### Saccharomyces cerevisiae

All budding yeast strains used in this study are SK1 derivatives. They were cultured under standard conditions at 30°C in YPD (yeast extract 1%, peptone 2%, dextrose 2%) either as liquid culture or on agar plates unless otherwise indicated. Details are specified in Table S2. All experiments were carried out in diploid strains.

### METHOD DETAILS

#### Strain generation

All strains used are SK1 derivatives. Details can be found in Table S2. The following alleles have been described previously: *ndt80Δ*^72,73^, *P_GPD_-GAL4(848)-ER*^55,72,74^, *P_GAL1_-CDC5-3HA*^72^, *P_GAL1_-ulp1*^*ΔN-GFP*44^, *P_CUP1_-CDC5*^70^, *CEN8-tdTomato*, *ARG4-yellow*, *THR1-cerulean*^45,50^, *mus81Δ*, *mlh1Δ*^48,50,75^, *ecm11^K5R^*, *ecm11^K101R^*,^42^ *NUP60-GFP*, *POM34-GFP*,^37^ *nup60Δ*, *nup60^9A^*, *P_CUP1_-CDC5-3xFLAG-10xHIS*, *P_CUP1_-CDC5^KD^-3xFLAG-10xHIS*,^32^ *ZIP1::GFP(700)*,^76^ *SPC42-mCherry*,^77^ *HTB1-mCherry*.^72^ *siz1Δ* and *siz2Δ* strains were a gift of the Gerben Vader lab. The *CLB2* promoter was used for meiosis-specific depletion of Cdc20 and Cdc5 protein.^62^ *ZIP1::mCherry2(700)* was engineered using CRISPR/Cas9 and recombineering in yeast. The resulting allele is based on *ZIP1::GFP(700)*^76^ where GFP is replaced by mCherry2. Gene deletions were introduced in the SK1 background by PCR-based amplification of the respective deletion cassettes from a yeast knockout collection or plasmids.^78,79^ For C-terminal epitope tagging of chromosomal genes, Myc9, Myc9-AID^(31-114)^, VH16 and GFP cassettes were amplified from plasmids by PCR as described.^80^ The plasmid containing the VH16 epitope tag was a gift from the Lackner lab and was described previously.^54^ The *Myc9-AID^(31-114)^* plasmid was a gift from H. Ulrich.^81^ The *ULP1^WT-GFP^* and *ulp1^ΔN-GFP^* alleles were obtained from F. Stutz.^53^ Strains carrying a copy of *ULP1^WT-GFP^* or *ulp1^ΔN-GFP^* in the *LEU2* locus were generated by cloning the construct into an integrative Yiplac128 vector^82^ and subsequent insertion at the *LEU2* locus by restriction digest (SacII) and transformation. Strains carrying the *ULP1^WT-GFP^* or *ulp1^ΔN-GFP^* at the endogenous locus were obtained by backcrossing strains YJM8422 and YJM8423 (both W303 background) to SK1 (8*x*). Strains carrying β-estradiol inducible *ULP1^WT-GFP^* were generated by replacing the endogenous promoter in a heterozygous strain (*ULP1* is an essential gene) with the *P_GAL_* cassette obtained from plasmid pYM-N23.^83^ Point mutations were introduced by mutagenic PCR. All plasmids and primers can be found in Table S2.

#### Meiotic time courses

Meiotic time courses were performed with diploid SK1 strains produced by mating the MATa and MAT*α* haploids, as previously described.^75,84^ In brief, cells were grown for 2 days at 30°C on YP_2% Glycerol_ (1% yeast extract, 2% peptone, 2% glycerol, 2% agar) plates and then amplified as a thin lawn on YPD plates (1% yeast extract, 2% peptone, 2% glucose, 2% agar). Cells were used to inoculate pre-sporulation medium YP_2% KAc_ (1% yeast extract, 2% peptone, 2% Kac) at OD_600_ = 0.3 and cultured for 14 h (25°C) or 11 h (30°C). Meiotic induction was initiated by switching cells to sporulation medium (SPM, 2% KAc) at OD_600_ = 3.5–4. The time of inoculation in SPM was defined as t = 0 h. Samples were collected at different intervals starting at 0 h until the time indicated in each experiment. Cell cycle stage distribution, release from G1 arrest, and entry into pre-meiotic S-phase were tracked by analyzing the cellular DNA content on a FACS Calibur cell sorter using propidium iodide (55 μg/mL) staining.

#### Meiotic time courses for live-cell imaging

Meiotic time courses were performed with diploid SK1 strains. Strains were first grown in YPD (1% yeast extract, 2% peptone, 2% glucose, 22.4 mg/L uracil, and 80 mg/L tryptophan) at room temperature (RT) or 30°C for ~20–24 h until saturation was reached (OD_600_ ≥ 10). Cultures were then back-diluted in BYTA (1% yeast extract, 2% bacto tryptone, 1% potassium acetate, and 50 mM potassium phthalate) to an OD_600_ = 0.25 and incubated at 30°C for ~14–20 h until reaching an OD_600_ ≥ 5. To induce sporulation, cells were pelleted, washed with MilliQ water and resuspended in sporulation media (SPM: 2% potassium acetate with 0.04 g/L adenine, 0.04 g/L uracil, 0.01 g/L histidine, 0.01 g/L leucine and 0.01 g/L tryptophan, adjusted to pH = 7) at an OD_600_ = 1.85. Cultures were maintained at 30°C for the duration of the experiment. During all steps, cultures were placed on a shaker in flasks that had 10*x* culture volume to ensure proper aeration.

#### Spore-autonomous fluorescence assay

The spore-autonomous fluorescence assay was used to determine the recombination rate in spores having completed meiosis as described before.^45^ In short, cells were prepared as for a time course and then sporulated for 48 h in SPM. 10 mL of cells were collected and washed once in SPM and then resuspended in 750 μL SPM. Cell suspension was sonicated 5 s at 10% amplitude on a Bandelin Sonoplus HD70 sonicator. 80 μL of cell suspension was added on a Poly-L-lysine (0.01%) coated standard microscopy slide and covered with a coverslip (50 × 25 mm). Excess liquid was removed by applying pressure. Slides were sealed and imaged on a DeltaVision Ultra Epifluorescence Microscope equipped with a UPlanXApo60*x*/1.42 Oil objective and a sCMOS camera controlled by AcquireUltra software (version 1.2.3). Experiments were carried out in triplicates, and 500 cells were quantified per replicate in Fiji.^71^ Data analysis and presentation were carried out in Microsoft Excel and GraphPad Prism.

#### Fluorescence light microscopy

For yeast whole cell immunofluorescence staining and yeast meiotic chromosome spread staining, published procedures were applied.^44,48,85^ For whole cell immunofluorescence imaging, cells were fixed for 45 min in 70% EtOH, washed in 1 mL 1xPBS, and resuspended in 500 μL PBS+DAPI (final concentration: 2.5 μg/mL). Cells were incubated for 5–10 min and washed three times in 1xPBS prior to imaging. Endogenous GFP-fluorescence and DAPI staining were assessed. For chromosome spreads, cells were collected and resuspended in 200 μL spheroplasting solution (2% (w/v) potassium acetate, 0.8 M sorbitol) and DTT at a final concentration of 10 mM was added for 15 min at 30°C. Subsequently cells were digested with Zymolyase 20T (0.25 mg/mL final concentration) for ~10 min. Spheroplasting was constantly monitored by mixing cells and sarcosyl (2% (w/v)) in a 1:1 ratio until cells lyse. The reaction was stopped by adding 400 μL ice-cold stop solution (0.1 M MES, 1 mM EDTA, 0.5 mM MgCl_2_, 1 M sorbitol, pH 6.4) and spheroplasts were collected by centrifugation (3 min, 700 rcf) and resuspended in 100 μL stop solution. For spreading, 20 μL of spheroplast solution was added to a glass slide and supplemented with 40 μL fixative (4% (w/v) paraformaldehyde, 3.4% (w/v) sucrose), 80 μL Lipsol (1% (w/v)), and another 80 μL fixative. Slides were dried overnight in a fume hood prior to staining. For staining, slides were washed in 1xPBS for 15 min and blocked 30 min in PBS containing 1% BSA. Slides were then incubated with 80 μL primary antibody in a humidity chamber for 4 h at RT. Slides were incubated with secondary antibody for 2 h at RT. After primary and secondary antibody incubation, slides were washed three times in 1xPBS for 5 min each wash. Slides were mounted with ProLong Diamond Antifade Mountant with DAPI (Invitrogen). Primary antibodies used were: rabbit anti-Zip1 (1:500 ^70^), rabbit anti-Ecm11 (1:600, a gift from A. Pichler), and mouse anti-Smt3 (1:500, Rockland 4F2.F5.G2, 200-301-42B). Secondary antibodies coupled to Alexa 555 and Alexa 488 were used for detection (1:300, Invitrogen). DNA was stained with 4′,6-diamidino-2-phenylindole (DAPI). Images were acquired on a DeltaVision Ultra epifluorescence microscope (GE Healthcare) with a 60 × 1.4NA Oil UPlanSApo or 100×1.4NA Oil UPlanSApo objective, using an sCMOS camera controlled by AcquireUltra software (version 1.2.3) or on a Zeiss Axio Imager M2 equipped with a 63×1.4 NA Oil Plan-Apochromat objective using a CoolSNAP HQ2 camera under the control of Zeiss ZEN blue 3.3. For the specific acquisition settings used in each experiment, see Table S2. In general, images were modified in Fiji for presentation using linear brightness and contrast adjustments. For polycomplex formation quantifications, >100 spreads were analyzed per genotype and replicate in Fiji.^71^ Statistical analyses were performed using Microsoft Excel and Prism.

#### Live-cell imaging

All live-cell fluorescent microscopy was performed with a DeltaVision Elite microscope (GE Healthcare) using a 60*x*/1.42 NA oil-immersion objective and a PCO Edge sCMOS camera. For the specific acquisition settings used in each experiment, see Table S2. The CellASIC ONIX2 platform (EMD Millipore) was used to maintain proper media flow and execute different treatment regimens. Cells at an OD_600_ = 1.85 in SPM were sonicated and loaded onto a microfluidics plate at 8 psi for 5 s. Conditioned SPM (prepared by filter-sterilizing a sporulating culture of a diploid yeast strain after ~5 h at 30°C) was flowed at 2 psi for the duration of the experiment to facilitate meiotic progression. All experiments were performed using Y04E microfluidic plates in a temperature-controlled chamber at 30°C.

#### Protein analyses

Samples were processed as described.^72^ In short, cell pellets were supplemented with glass beads and disrupted in 10% TCA using a FastPrep-24 (MP Biomedicals) running two cycles of 40 s (6.5 m/s). The protein precipitates were resuspended in 2*X* NuPAGE sample buffer and neutralized with 1 M Tris base at a 2:1 ratio, boiled at 95°C for 10 min, and cleared by centrifugation at full speed for another 10 min. Preceding sample loading, concentration was assessed. For immunoprecipitation, 50 mL sample of meiotic cultures (OD_600_ = 3.5) were supplemented with PMSF/DMSO (2 mM final) upon collection. Cells were lysed in buffer B (50 mM HEPES/KOH pH7.4, 100 mM β-glycerophosphate, 5 mM MgOAc, 0.1% Triton X-100, 10% Glycerol, 20 mM NaF). 1 mM DTT, 1 mM PMSF and protease inhibitor tablets (Roche, 1 Tablet/50 mL) were freshly added. Lysates were cleared and adjusted to equal protein concentrations. Myc-tagged proteins were captured using anti-Myc agarose beads (9E10, Cancer Research UK) while rotating for 1 h at 4°C. Agarose beads were split in 4 equal parts for phosphatase treatment after several rounds of washes with increasing salt (2 × 70 mM, 1 × 150 mM, 1 × 200 mM, 2 × 70 mM KAc). Phosphatase treatment was carried out using 4 conditions: 1) PMP buffer and MnCl_2_; 2) PMP buffer and MnCl_2_; 3) PMP buffer, MnCl_2_ and λ-phosphatase; 4) PMP buffer, MnCl_2_ and heat-inactivated λ-phosphatase. Conditions 2–4 were incubated 15 min at 30°C. Samples were washed twice prior to analysis by western blotting. Protein samples were separated on NuPAGE 3–8% Tris-Acetate (Invitrogen) or NuPAGE 4–12% Bis-Tris gels using matching running buffers. Proteins were transferred onto Amersham Hybond 0.45 μm PVDF membranes. For immunoblotting the following antibodies were used: rabbit anti-Myc HRP conjugated (1:15000, ab1326 Abcam, RRID:AB_299800), mouse anti-HA (1:2500, Clone 16B12 Biolegend, RRI-D:AB_291262), mouse anti-GFP (1:2500 11814460001 Roche, RRID:AB_390913), mouse anti-Cdc5 (1:2500 MM-0192-1-100 Méd-iMabs), rabbit anti-Ecm11 (1:5000, a gift from A. Pichler), rabbit anti-Smt3 (1:5000, gift from A. Pichler), and rabbit anti-Crm1 (1:5000, gift Weis lab^69^). The following secondary antibodies were used: 1:5000 goat anti-mouse immunoglobulin conjugated to HRP (P0447 Agilent, RRID:AB_2617137) and 1:5000 swine anti-rabbit immunoglobulin HRP conjugated (P0339 Agilent, RRID:AB_2617141). Signals were quantified in Fiji^71^ and plotted in Excel and GraphPad Prism.

#### FACS

Cells from a meiotic time course were sampled at different timepoints post-SPM inoculation. 1 mL was collected, centrifuged at 20000 *x g* and supernatant was aspirated. Pellet was resuspended in 1 mL EtOH 70% and stored at 4°C overnight. Cells were washed 2*x* in 50 mM Tris-HCl and then resuspended in 500 μL 50 mM Tris-HCl supplemented with 2 μL RNAse A (100 mg/mL) to digest RNA for 4 h at 37°C. Cells were washed once in FACS buffer (200 mM Tris-HCl pH 7.5, 211 mM NaCl, 78 mM MgCl_2_) and sonicated in FACS buffer supplemented with 55 μg/mL propidium iodide. 60 μL of sample in 1 mL 50 mM Tris-HCl pH 7.5 was used to assess DNA content on a FACS Calibur machine (Beckton Dickinson) running FACSDiva software. For each sample, >25000 cells were analyzed. Profiles were analyzed using FlowJo Version 10.9.0 (BD Biosciences).

#### Spore viability assay

Yeast strains were freshly thawed on YPD plates and grown at 30°C for 24 h. Cells were subsequently transferred to SPM plates and incubated for 24 h at 30°C to induce sporulation. Prior to dissection, spore wall was digested for 10 min using 20T Zymolyase (1:10 dilution in H_2_O from 1 mg/mL stock). 20 μL of digest mix was plated on one side of a YPD plate. Individual spores of one tetrad were relocated on the YPD plate using a Microdissector (Singer MSM400). Cells were grown at 30°C for 48 h. Colony growth was assessed. A total of 432 tetrads from 6 replicates (72 tetrads each) were analyzed per genotype.

### QUANTIFICATION AND STATISTICAL ANALYSIS

Statistical analyses for spore viability and spore-autonomous fluorescence assays were performed using Microsoft Excel and Prism. For multiple comparisons, analysis of variance (one-way ANOVA) was performed with Prism, followed by a correction for multiple comparisons using statistical hypothesis testing (Dunnett’s and Tukey’s test). For pairwise comparisons, two-tailed unpaired t-tests were used. The full summary statistics and test output for each experiment can be found in Table S1. For all experiments, asterisks represent the following *p* values: *p < 0.05; **p < 0.01; ***p < 0.001; ****p < 0.0001; ns = non-significant.

#### Live-cell image quantification and statistics

All live-cell image analysis was performed in Fiji (RRID:SCR_002285).^71^ Maximum z-projection and single z-slices are shown for each image and were modified for presentation using linear brightness and contrast adjustments in Fiji.

Quantification of meiotic progression in strains expressing Zip1-GFP Spc42-mCherry or Zip1-mCherry Spc42-mCherry was performed using the following definitions. “Zip1 appearance” was defined as the first time point when Zip1-GFP or Zip1-mCherry signal was distinguishable from background cellular fluorescence. “Zip1 chromosomal localization” was defined as the first time point when Zip1-GFP or Zip1-mCherry signal exhibited punctate or patterned – rather than diffuse nucleoplasmic – localization. “Zip1 disappearance” was defined as the first time point when Zip1-GFP or Zip1-mCherry signal was lost to a level indistinguishable from background cellular fluorescence. “Anaphase I” was defined as the first time point when two Spc42-mCherry puncta moved rapidly away from each other, consistent with an elongating spindle. “Anaphase II” was defined as the first time point when four Spc42-mCherry puncta moved rapidly away from each other, consistent with two elongating spindles. Cells completing a final mitotic division at the start of image acquisition were not included in timing quantification.

Quantification of nuclear envelope intensity for fluorescently tagged proteins was performed similarly to King et al., 2023.^32^ In brief, individual cells were cropped from deconvolved images with background signal subtracted using a rolling ball method with a radius of 15 pixels. For the time points of interest, the individual z-slices containing the middle of the nucleus were selected, and a nuclear mask was generated using the Htb1-mCherry signal. This nuclear mask was eroded and then dilated to generate masks for the nucleoplasmic and nuclear envelope regions, respectively. Area-normalized nuclear envelope intensity was then calculated by dividing total nuclear envelope intensity by nuclear envelope area. These values were then normalized to the mean value of the indicated control of each experiment and used for downstream analysis.

Plotting and statistics were conducted using either GraphPad Prism (v10) or R 4.3.2 (R Core Team 2023). The full summary statistics and statistical test output for each experiment are provided in Table S1 and all source codes used for analysis are provided at https://doi.org/10.5281/zenodo.20317646. For all experiments, asterisks represent the following *p* values: * *p* < 0.05; ** *p* < 0.01; *** *p* < 0.001; **** *p* < 0.0001.

## Supplementary Material

1

2

3

4

5

6

7

8

9

Supplemental information can be found online at https://doi.org/10.1016/j.celrep.2026.117539.

## Figures and Tables

**Table:** KEY RESOURCES TABLE

REAGENT or RESOURCE	SOURCE	IDENTIFIER
Antibodies		
Mouse anti-GFP	Roche	Cat#11814460001; RRID:AB_390913
Mouse anti-HA clone 16B12	BioLegend	Cat#MMS-101P-200; RRID:AB_291262
Mouse anti-Cdc5	Médimabs	Cat#MM-0192-1-100
Rabbit anti-Myc HRP	Abcam	Cat#ab1326; RRID:AB_299800
Rabbit anti-Crm1	K. Weis (ETH Zurich)	not commercial; no RRID. Onischenko et al. 2009^69^
Rabbit anti-Zip1	J. Matos (University of Vienna)	Not commercial; no RRID Grigaitis et al., 2020^70^
Rabbit anti-Ecm11	A. Pichler (MPI Freiburg)	Not commercial; no RRID Gift from A. Pichler
Rabbit anti-Smt3	A. Pichler (MPI Freiburg)	Not commercial; no RRID Gift from A. Pichler
Mouse anti-Smt3 clone 4F2.75.G2	Rockland	Cat#200-301-42B
Goat anti-Mouse IgG (HRP)	Agilent	Cat#P0447; RRID:AB_2617137
Swine anti-Rabbit IgG (HRP)	Agilent	Cat#P0399; RRID:AB_2617141
Donkey anti-rabbit Alexa Fluor 488	Thermo Fisher Scientific	Cat#A-21206; RRID:AB_2535792
Donkey anti-mouse Alexa Fluor 555	Thermo Fisher Scientific	Cat#A-31570; RRID:AB_2536180
Chemicals, peptides, and recombinant proteins		
Bio-Rad Protein Assay	Bio-Rad	Cat#5000006
NuPAGE^™^ sample buffer	Thermo Fisher Scientific	Cat#NP0008
Tris Acetate running buffer (20*X*)	Thermo Fisher Scientific	Cat#LA0041
MES running buffer (20*X*)	Thermo Fisher Scientific	Cat#NP0002
BioRad Precision Plus Protein Dual Color Standard	BioRad	Cat#1610374
PageRuler Prestained Protein Ladder	Thermo Fisher Scientific	Cat#26616
RNase A	Roche	Cat#10109169001
Propidium Iodide	Sigma Aldrich	Cat#81845
ProLong^™^ Diamond Antifade Mountant with DAPI	Thermo Fisher Scientific	Cat#P36962
Zymolyase 20T	Seikagaku Biobusiness	Cat#120491
Zymolyase 100T	Seikagaku Biobusiness	Cat#120493-1
Deposited data		
Raw data for Figures 1, 2, 3, 4, and S1-S4	This study	Mendeley: Wettstein, Rahel; King, Grant A. (2026), “Synaptonemal Complex SUMOylation is maintained by Nup60-dependent docking of Ulp1 at the nuclear periphery”, Mendeley Data, V1, https://doi.org/10.17632/7x7vzf9y8b.1
Analysis Scripts for Figures 1, 2, 4, S1, and S3	This study	Github: https://doi.org/10.5281/zenodo.20317646
Experimental models: Organisms/strains		
All strains used in this study are listed in Table S2	This study	N/A
OligonucleotidesRecombinant DNA		
All primers used in this study are listed in Table S2	This study	N/A
Recombinant DNA		
All plasmids used in this study are listed in Table S2	This study	N/A
Software and algorithms		
Fiji (Fiji Is Just ImageJ)	Schindelin et al.^71^	https://fiji.sc/
Prism 9	GraphPad	https://www.graphpad.com/
FlowJo v10	Becton Dickinson	https://www.bdbiosciences.com/en-ch/products/software/flowjo-v10-software
R 4.3.2	R Core Team	https://cran.r-project.org/bin/windows/base/old/
Other		
NuPAGE^™^ Novex^™^ 3–8%, Tris-Acetate Midi gels	Thermo Fisher Scientific	Cat#WG1602BOX
NuPAGE^™^ Novex^™^ 4–12% Bis-Tris Midi gels	Thermo Fisher Scientific	Cat#WG1402BOX
Amersham^™^ Hybond^™^ P 0.45 PVDF blotting membrane	Cytiva	Cat#GE10600023
DeltaVision Ultra Epifluorescence Microscope	Image Solutions	N/A
ChemiDoc MP	Bio-Rad	N/A
FastPrep-24	MP Biomedicals	N/A
FACSCalibur	Becton Dickinson	N/A
CellASIC ONIX2 platform	EMD Millipore	Cat#CAX2-S0000

## Highlights

- The NPC basket spatially restricts SUMO proteolysis during meiosis
- Ulp1 mislocalization reduces Ecm11 SUMOylation and impairs crossover control
- Cdc5 regulates the meiotic SUMO landscape by targeting SUMO ligases and proteases
