# Supplementary material for: Synaptonemal complex SUMOylation is maintained by Nup60-dependent docking of Ulp1 at the nuclear periphery
Source: Cell Rep. Author manuscript; Available in PMC 2026 Jul 20. (PMC13384433; doi:10.1016/j.celrep.2026.117539)
Supplement: 1 [file NIHMS2190754-supplement-1.pdf]

Cell Reports, Volume *45*

## Supplemental information

# Synaptonemal complex SUMOylation is maintained by Nup60-dependent docking of Ulp1 at the nuclear periphery

**Rahel Wettstein, Grant A. King, Adrian Henggeler, Madison E. Walsh, Cyrus T. Ruediger, Elçin Ünal, and Joao Matos**

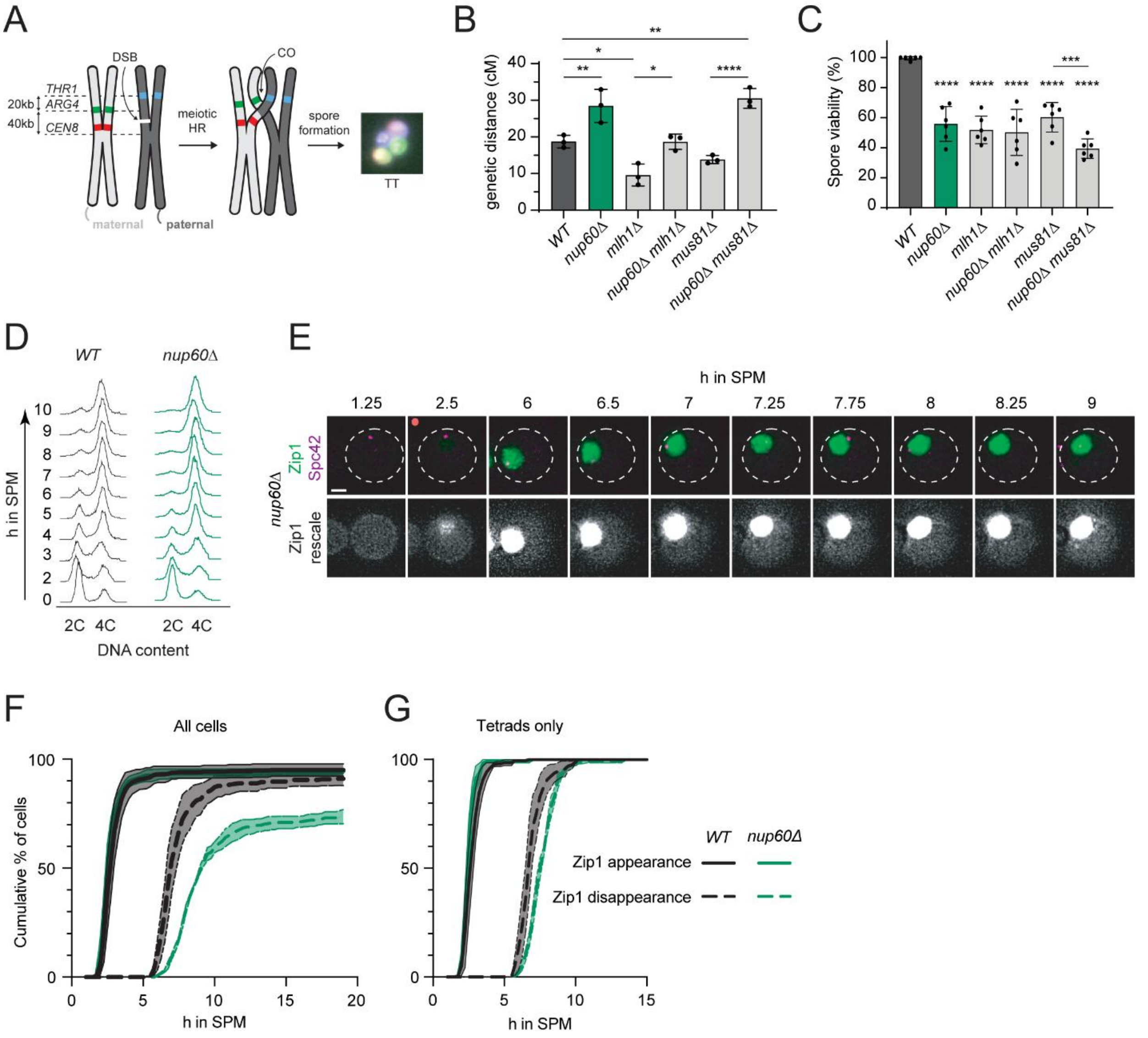


**Figure S1. Nup60 supports chromosome synapsis** (Related to Figure1, Table S1)**. (A)** Schematic representation of the spore-autonomous fluorescence system located on chromosome VIII derived from Arter et al. 2018[1,2]. Three fluorescent markers are introduced on chromosome VIII in a heterozygous manner. The distal fluorophores are separated by 60 kb. Fluorophores are expressed upon spore wall formation. **(B)** Spore-autonomous fluorescence analysis of recombination frequency in strains with the indicated genotypes. The experiment was carried out in triplicates (n = 500 cells per genotype and replicate). Bar graph represents mean+/-SD. **(C)** Spore viability assay of strains with the indicated genotypes. The experiment was carried out in six replicates (n=72 spores per genotype and replicate). Bar graph represents mean+/-SD. **(D)** DNA content of meiotic samples measured by FACS for strains with the indicated genotypes. Samples were collected at regular time intervals after transfer to sporulation medium (SPM) and compared. **(E)** Montages from live-cell imaging, depicting meiotic progression in strains with the indicated genotype. Meiotic progression was monitored as in Figure 1D. Representative images of a cell failing to disassemble Zip1 are shown. Red dot indicates time point scored as Zip1 appearance. Scale bar, 2 µm **(F-G)** Kinetics of Zip1 appearance (solid line) and disappearance (dashed line) for strains of the indicated genotypes quantified from live-cell imaging. Lines represent the mean and shaded ranges represent the standard error of the triplicates. **(F)** All cells were considered (n≥100 cells per replicate). **(G)** Only cells which formed tetrads by the end of the imaged timeframe were considered (n≥40 tetrads per

replicate). Statistical testing: **p*<0.05; ***p*<0.01; ****p*<0.005; *****p*<0.001; ns=non-significant by Tukey's multiple comparisons test (B and C). *p* values in Table S1.

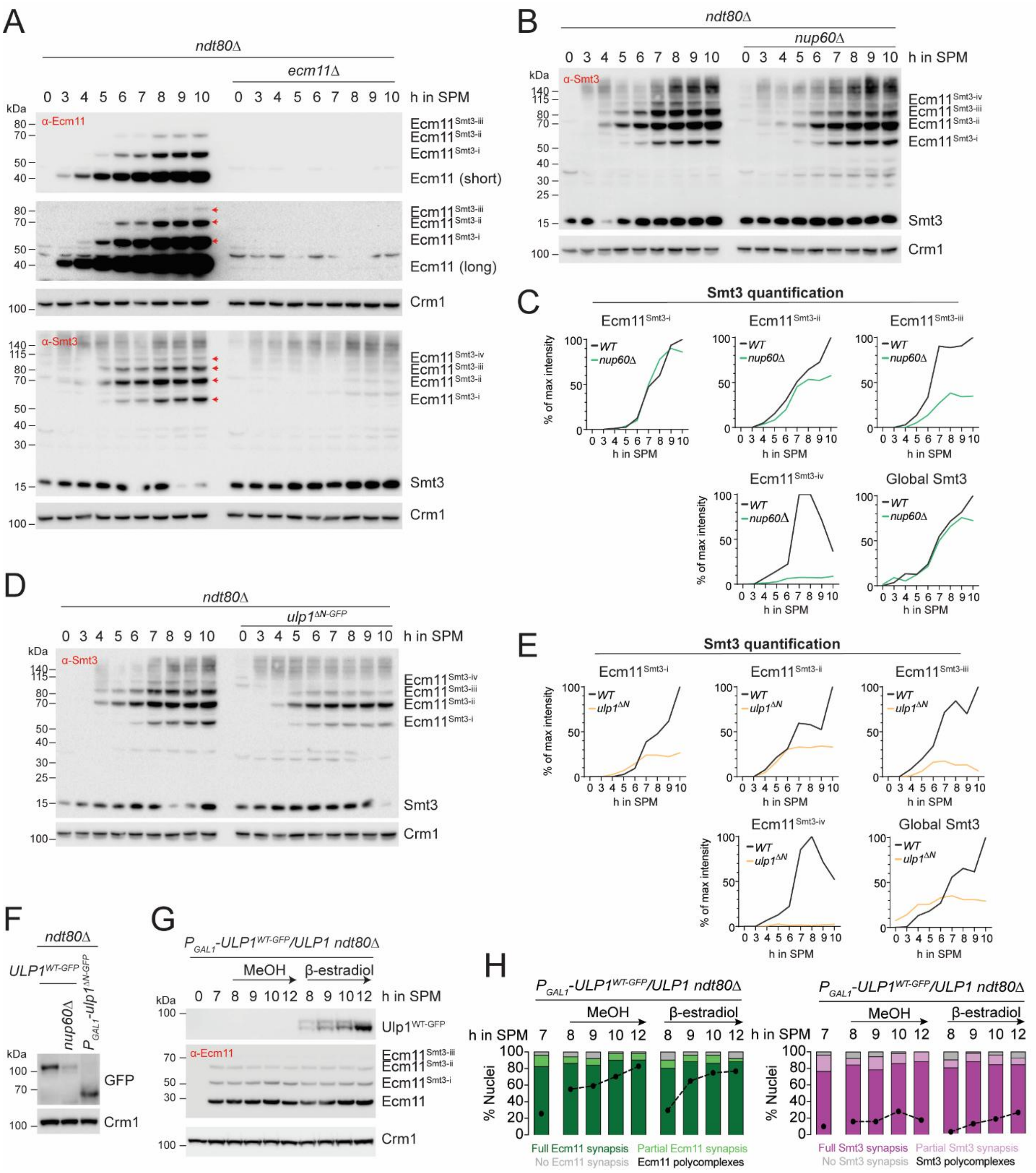

**Figure S2. Anchoring the SUMO protease Ulp1 to the NPC affects global SUMOylation in pachytene cells** (Related to Figure 3)**. (A)** Westen blot analysis of strains with the indicated genotypes in meiosis. Samples were collected at specified times in SPM. Anti-Ecm11 and anti-Smt3 antibodies were used to monitor Ecm11 and SUMO levels respectively. Crm1 served as normalization control. SUMO modifications specific for Ecm11 are indicated by red arrowheads. **(B)** Western blot analysis of SUMOylation for strains with the indicated genotypes in meiosis. Samples were collected at specified times in SPM. Anti-Smt3 antibody was used to detect SUMOylation. Ecm11$^{Smt3\text{-}i\text{-}iv}$ is used to indicate different modified versions of Ecm11 protein detected by the Smt3 antibody. Crm1 was used as normalization control. Same samples as in Figure 3A were used. **(C)** Quantification of (B) separated by different SUMO-modified versions of Ecm11.

Crm1 signal was used for normalization of Smt3 signal. **(D)** Western blot analysis of SUMOylation for strains with the indicated genotypes in meiosis. Samples were collected at specified times in SPM. Anti-Smt3 antibody was used to detect SUMOylation. Ecm11$^{Smt3\text{-}i\text{-}iv}$ is used to indicate increasingly modified versions of Ecm11 protein detected by the Smt3 antibody. Crm1 was used as normalization control. Same samples as in Figure 3C were used. **(E)** Quantification of (D) separated by different modified versions of Ecm11. Crm1 signal was used for normalization of Smt3 signal. **(F)** Western blot analysis of Ulp1 and ulp1$^{\Delta N}$ protein in *ndt80Δ* or *ndt80Δ nup60Δ* background. Ulp1 was detected using an anti-GFP antibody. Crm1 served as normalization control. **(G)** Western blot analysis of Ecm11 and Ulp1$^{WT\text{-}GFP}$ in extracts from cells with the indicated genotype at specified times in SPM. Ulp1$^{WT\text{-}GFP}$ was induced by addition of 2 μM β-estradiol (or MeOH as control) at 7 h in SPM. Crm1 served as normalization control. Control for the experiment shown in Figure 3E. **(H)** Quantification of the extent of Ecm11 and Smt3 synapsis from the experiment in (G), showing fully synapsed (dark green/magenta), partially synapsed (bright green/lilac), and non-synapsed SCs (grey). Black dotted line indicates fraction of nuclei with a polycomplex. 50 nuclei were analyzed per time point.

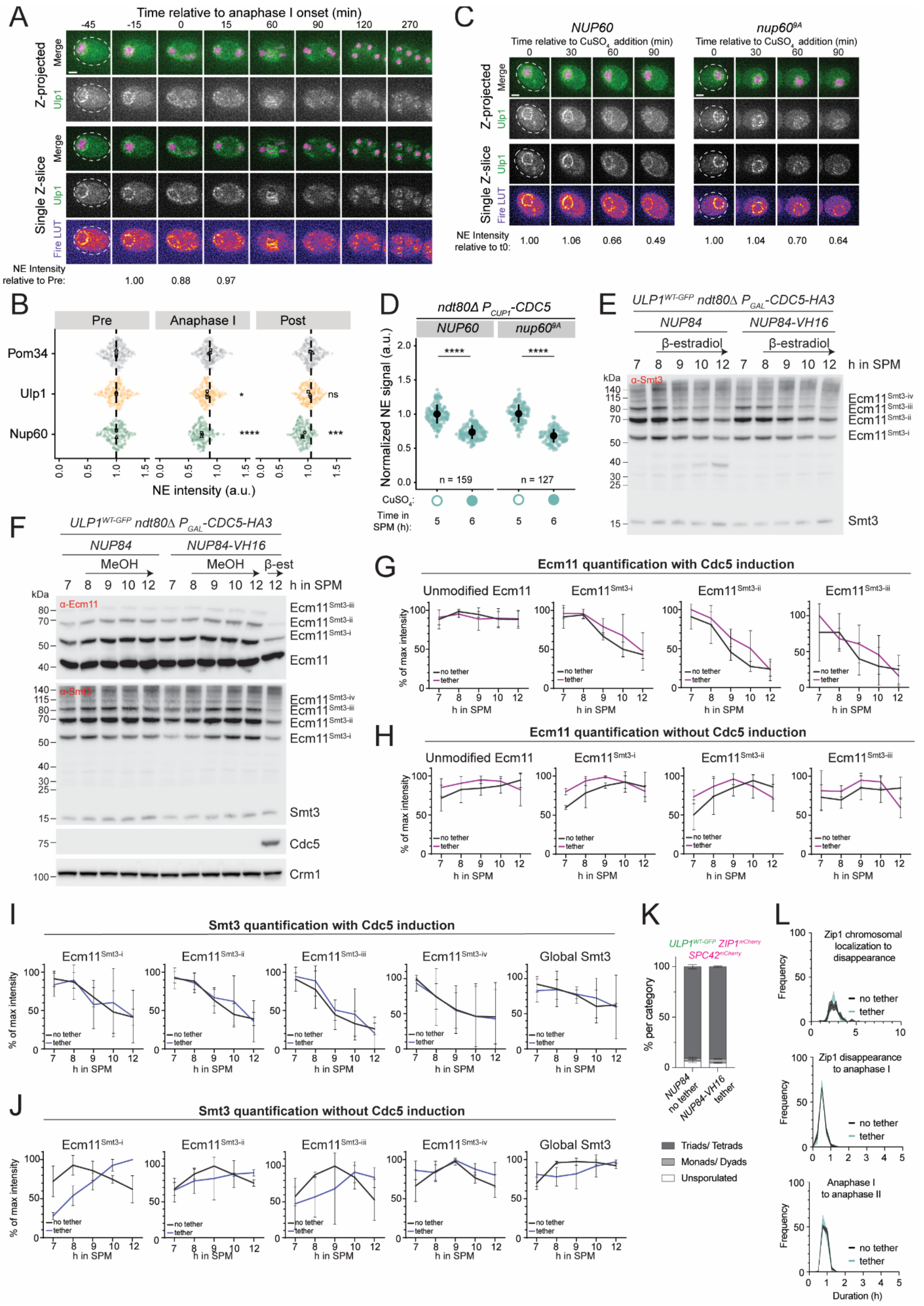
A
Time relative to anaphase I onset (min)
-45 -15 0 15 60 90 120 270
Z-projected
Merge
Ulp1
Single Z-slice
Fire LUT
NE Intensity relative to Pre: 1.00 0.88 0.97
B
Pre
Anaphase I
Post
Pom34
Ulp1
Nup60
NE intensity (a.u.)
C
NUP60
nup60-9A
Time relative to CuSO4 addition (min)
NE Intensity relative to t0: 1.00 1.06 0.66 0.49 1.00 1.04 0.70 0.64
D
ndt80Δ PCUP1-CDC5
Normalized NE signal (a.u.)
n = 159
n = 127
CuSO4:
Time in SPM (h):
E
ULP1WT-GFP ndt80Δ PGAL-CDC5-HA3
NUP84
NUP84-VH16
β-estradiol
h in SPM
α-Smt3
Smt3
F
MeOH
β-est
α-Ecm11
Ecm11
Cdc5
Crm1
G
Ecm11 quantification with Cdc5 induction
Unmodified Ecm11
% of max intensity
h in SPM
no tether
tether
H
Ecm11 quantification without Cdc5 induction
I
Smt3 quantification with Cdc5 induction
Global Smt3
J
Smt3 quantification without Cdc5 induction
K
ULP1WT-GFP ZIP1mCherry SPC42mCherry
% per category
NUP84 no tether
NUP84-VH16 tether
Triads/ Tetrads
Monads/ Dyads
Unsporulated
L
Zip1 chromosomal localization to disappearance
Zip1 disappearance to anaphase I
Anaphase I to anaphase II
Frequency
Duration (h)

**Figure S3. Cdc5 regulates SUMOylation by coordinately targeting SUMO ligases and SUMO proteases** (Related to Figure 4, Table S1, Video S3)**.** **(A)** Montage from live-cell imaging of strains progressing through meiosis with the indicated genotypes using Ulp1$^{WT\text{-}GFP}$ and histone Htb1$^{mCherry}$ fluorescent tags. Scale bar, 2 μm. The full movie can be found in Video 3. NE: nuclear envelope. **(B)** Quantification of Ulp1$^{WT\text{-}GFP}$ localization to the nuclear periphery during meiosis I, corresponding to (A). Quantification of strains with Pom34$^{GFP}$ – a marker that remains at the nuclear periphery – or Nup60$^{GFP}$ – a marker that detaches from the nuclear periphery – were included as controls. “Anaphase I” was defined as the first timepoint when the spherical Htb1$^{mCherry}$ mass exhibited a distortion consistent with chromosome segregation, with “Pre” and “Post” defined as 15 minutes before or after this event. Nuclear envelope intensity values were normalized to the average nuclear envelope intensity at the “Pre” timepoint (-15 min) for each marker. Only cells that became tetrads were considered (n≥50 tetrads per replicate). Mean values for individual replicates are shown in black. Pom34$^{GFP}$ served as the reference for statistical testing. **(C)** Montages from live-cell imaging of strains with the indicated genotypes using Ulp1$^{WT\text{-}GFP}$ and histone Htb1$^{mCherry}$ fluorescent tags. *$P_{CUP1}$-CDC5-3xFLAG-10xHis* expression was induced at 5 h in SPM with 50 μM $CuSO_4$. Scale bars, 2 μm. **(D)** Quantification of Ulp1 detachment in (C). Nuclear envelope intensity values were normalized to the average nuclear envelope intensity for *NUP60* cells at the pre-induction time point (5 h in SPM). Post-induction (6 h in SPM) values were compared to pre-induction (5 h in SPM) values for each treatment regimen to assess statistical significance. Sample size (*n*) is indicated in the panel. **(E)** Westen blot analysis of strains with the indicated genotypes in meiosis. *$P_{GAL1}$-CDC5-3HA* expression was induced by addition of 2 μM β-estradiol at 7 h in SPM. Samples were collected at 1-hour intervals after β-estradiol addition. Anti-Smt3 antibody was used to detect SUMOylation. Ecm11$^{Smt3\text{-}i\text{-}iv}$ is used to indicate different modified versions of Ecm11 protein detected by the Smt3 antibody. Crm1 served as normalization control. Same samples as in Figure 4F are used. **(F)** Westen blot analysis of strains with the indicated genotypes in meiosis. MeOH was added to the culture at 7 h in SPM for uninduced control of *$P_{GAL1}$-CDC5-3HA* expression. Samples were collected in 1-hour intervals starting from time of MeOH addition. Anti-HA antibody was used to detect Cdc5. Anti-Ecm11 antibody and anti-Smt3 antibody were used to detect Ecm11 and SUMO, respectively. Crm1 served as normalization control. Blot is the uninduced control for Figure 4F. **(G)** Quantification of Ecm11 signal in Figure 4F separated by different modified versions of Ecm11. Crm1 signal was used for normalization of Ecm11 signal. Data shown represents mean±SD from three independent experiments. **(H)** Quantification of Ecm11 signal in (F) separated by different modified versions of Ecm11. Crm1 signal was used for normalization of Ecm11 signal. Data shown represents mean±SD from three independent experiments. **(I)** Quantification of Smt3 signal in (E) separated by different modified versions of Ecm11. Crm1 signal was used for normalization of Smt3 signal. Data shown represents mean±SD from three independent experiments. **(J)** Quantification of Smt3 signal in (F) separated by different modified versions of Ecm11. Crm1 signal was used for normalization of Smt3 signal. Data shown represents mean±SD from three independent experiments. **(K-L)** Meiosis was assessed in *WT* (no tether) and *NUP84-VH16* (tether) strains with Ulp1$^{WT\text{-}GFP}$, synaptonemal complex component Zip1$^{mCherry}$, and spindle pole body component Spc42$^{mCherry}$. **(K)** Sporulation efficiency in flask measured after 24 h in SPM. At least 300 cells were counted for each replicate, with error bars representing the standard error measurement for triplicates. **(L)** Meiotic progression assessed by imaging cells every 15 min after 1-1.5 h in SPM. Duration of Zip1 presence on chromosomes (top), duration from Zip1 disappearance to anaphase I (middle), and duration from anaphase I to anaphase II (bottom) were calculated. Only cells which formed tetrads in the imaged time span were considered for analysis (n≥50 tetrads per replicate). Lines and shaded ranges represent the mean+/-SE of the triplicates. Statistical testing: *$p$<0.05; **$p$<0.01; ***$p$<0.005; ****$p$<0.001; ns=non-significant by Tukey’s multiple comparisons test comparing the means of individual replicates (B) or a Wilcoxon signed-rank test (D). $p$ values in Table S1.

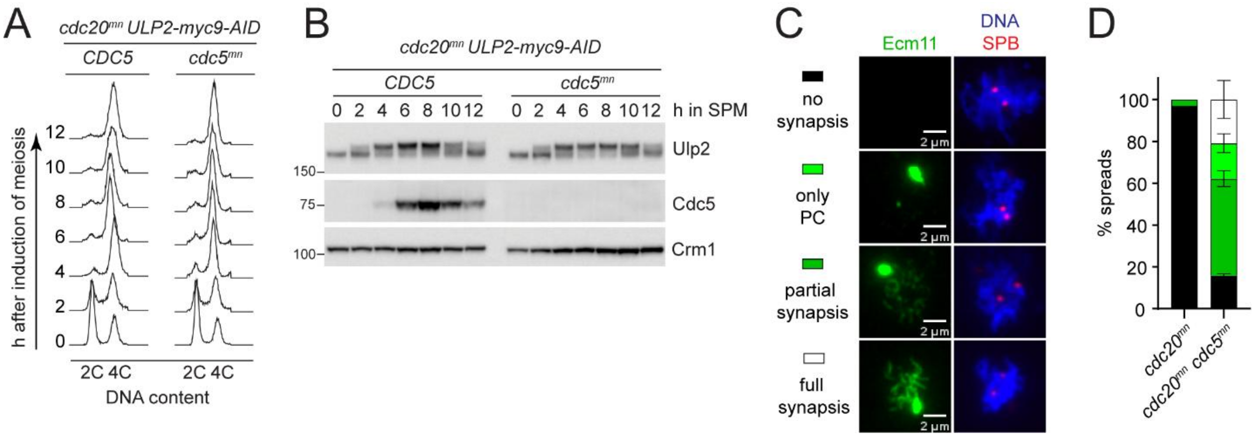


**Figure S4. SUMOylated Ecm11 and global SUMOylation in meiosis increases in the absence of Cdc5** (Related to Figure 4)**. (A)** Measurement of DNA content by FACS of the indicated genotypes. A representative experiment is shown from duplicates. **(B)** Western blot analysis of Ulp2 protein in strains of the indicated genotypes. Samples were collected at 2-hour intervals. Ulp2 protein was detected using an anti-Myc antibody. Cdc5 was detected with an anti-Cdc5 antibody. Crm1 served as normalization control. **(C)** Metaphase spreads co-stained for Ecm11, DAPI and α-tubulin (representing SPB). Shown are representative images of metaphase spreads with different levels of Ecm11 loading onto chromosomes. **(D)** Classification of metaphase spreads into categories defined in (C) for the indicated genotypes. Experiment was performed in duplicates (n≥30 spreads per replicate). Stacked bar graph represents mean +/- upper and lower limits. Scale bar, 2 µm. PC=polycomplex, SPB=spindle pole body.

## SUPPLEMENTAL REFERENCES

[1.] Arter, M., Hurtado-Nieves, V., Oke, A., Zhuge, T., Wettstein, R., Fung, J.C., Blanco, M.G., and Matos, J. (2018). Regulated Crossing-Over Requires Inactivation of Yen1/GEN1 Resolvase during Meiotic Prophase I. Dev Cell *45*, 785-800 e786. 10.1016/j.devcel.2018.05.020.

[2.] Thacker, D., Lam, I., Knop, M., and Keeney, S. (2011). Exploiting spore-autonomous fluorescent protein expression to quantify meiotic chromosome behaviors in Saccharomyces cerevisiae. Genetics *189*, 423-439. 10.1534/genetics.111.131326.
